# Melanoma Spheroids Grown Under Neural Crest Cell Conditions Are Highly Plastic Migratory/Invasive Tumor Cells Endowed with Immunomodulator Function

**DOI:** 10.1371/journal.pone.0018784

**Published:** 2011-04-15

**Authors:** Kiran Ramgolam, Jessica Lauriol, Claude Lalou, Laura Lauden, Laurence Michel, Pierre de la Grange, Abdel-Majid Khatib, Fawzi Aoudjit, Dominique Charron, Catherine Alcaide-Loridan, Reem Al-Daccak

**Affiliations:** 1 Unité Mixte de Recherche Scientifique (UMRS) 940, Institut National de la Santé et de la Recherche Médicale (INSERM), Institut Universitaire d'Hématologie, Université Paris-Diderot Paris 7, Hôpital St Louis, Paris, France; 2 Centre National de Recherche Scientifique (CNRS) UMRS 7592, Institut Jacques Monod, Université Paris-Diderot Paris 7, Paris, France; 3 UMRS976, INSERM, Université Paris-Diderot Paris 7, Hôpital St Louis, Paris, France; 4 GenoSplice Technology, Institut Universitaire d'Hématologie, Hôpital St Louis, Paris, France; 5 Centre de Recherche en Rhumatologie et Immunologie, Centre de Recherche du Centre Hospitalier Universitaire de Québec (CRCHUQ), Laval University, Quebec, Canada; 6 Centre d'Investigations Biomédicales-Hématologie, Oncologie et Greffes (CIB-HOG), Hôpital St Louis, Assistance Publique des Hôpitaux de Paris (AP-HP), Paris, France; Dana-Farber Cancer Institute, United States of America

## Abstract

**Background:**

The aggressiveness of melanoma tumors is likely to rely on their well-recognized heterogeneity and plasticity. Melanoma comprises multi-subpopulations of cancer cells some of which may possess stem cell-like properties. Although useful, the sphere-formation assay to identify stem cell-like or tumor initiating cell subpopulations in melanoma has been challenged, and it is unclear if this model can predict a functional phenotype associated with aggressive tumor cells.

**Methodology/Principal Findings:**

We analyzed the molecular and functional phenotypes of melanoma spheroids formed in neural crest cell medium. Whether from metastatic or advanced primary tumors, spheroid cells expressed melanoma-associated markers. They displayed higher capacity to differentiate along mesenchymal lineages and enhanced expression of SOX2, NANOG, KLF4, and/or OCT4 transcription factors, but not enhanced self-renewal or tumorigenicity when compared to their adherent counterparts. Gene expression profiling attributed a neural crest cell signature to these spheroids and indicated that a migratory/invasive and immune-function modulating program could be associated with these cells. In vitro assays confirmed that spheroids display enhanced migratory/invasive capacities. In immune activation assays, spheroid cells elicited a poorer allogenic response from immune cells and inhibited mitogen-dependent T cells activation and proliferation more efficiently than their adherent counterparts. Our findings reveal a novel immune-modulator function of melanoma spheroids and suggest specific roles for spheroids in invasion and in evasion of antitumor immunity.

**Conclusion/Significance:**

The association of a more plastic, invasive and evasive, thus a more aggressive tumor phenotype with melanoma spheroids reveals a previously unrecognized aspect of tumor cells expanded as spheroid cultures. While of limited efficiency for melanoma initiating cell identification, our melanoma spheroid model predicted aggressive phenotype and suggested that aggressiveness and heterogeneity of melanoma tumors can be supported by subpopulations other than cancer stem cells. Therefore, it could be constructive to investigate melanoma aggressiveness, relevant to patients and clinical transferability.

## Introduction

Melanoma represents one of the most aggressive malignancies with a high tendency to invade secondary sites. Less than 10% of patients presenting metastasis survive over one year due to the lack of efficient therapy. Numerous novel therapeutic protocols have been developed but, display little improvement over existing chemotherapy protocols. Melanoma presents a variety of phenotypic and behavioral features. Melanoma tissues have various morphologies and immunohistochemical staining of melanoma lesions for specific markers often leads to heterogeneous results [1], [2], [3]. Therefore, one explanation for the therapeutic failures might reside in the selective targeting of melanoma cells due to their heterogeneity.

The heterogeneity is illustrated by the existence of multi subpopulations within a melanoma tumor. Several gene expression studies suggested that there are specific transcriptional signatures that delineate melanoma cells subpopulations [4], [5], [6]. Importantly, *in vivo* studies showed that these specific transcriptional signatures are linked and reversible given appropriate signals and microenvironment cues, which suggested that melanoma progression is associated with transcription signature plasticity [7]. These studies thus, provide a rational context for melanoma cells heterogeneity. One noteworthy example of melanoma cell plasticity is the ability of aggressive melanoma cells to adopt endothelial-like properties and mimic embryonic vasculogenic networks [8]. Another example is the observation that placing metastatic melanoma cells in a chick embryo microenvironment or in zebra fish embryos suppresses their tumorigenic phenotype and reprograms the metastatic phenotype of a subpopulation of tumor cells [9], [10]. The tumorigenic phenotype of aggressive melanoma cells is also suppressed when placed in human embryonic stem cell microenvironment [11].

Interestingly, growing melanoma cells from metastatic or primary lesions as spheroids in human embryonic or neural stem cells medium further supported the notion of plasticity by defining subpopulations capable of self-renewal and differentiation into multiple lineages [12], [13], [14]. These spheroid cells displayed enhanced tumorigenicity and were enriched with melanoma initiating or melanoma cancer stem cells (CSC) [12], [13], [14]. Growth of tumor cells in three-dimensional multicellular tumor spheroid cultures has been considered to replicate some of the complex features of *in situ* solid tumors. Indeed, tumor spheroid cultures are a rather classical approach to obtain and maintain the functional phenotype of human tumors, and thus represent a more physiologically relevant *in vitro* model of tumors. Yet the utility of sphere-formation under stem cell conditions as a surrogate tool for CSC identification in human melanoma has been recently challenged given the lack of clear and consistent correlations between melanoma initiating or CSC phenotype with sphere-forming ability [15]. However, sphere-formation of murine melanoma cells on non-adhesive substrates such as polyHema was suggested as an appropriate model to mimic the different growth and progression patterns obtained *in vivo* [16], [17], [18]. In addition, the gene expression profile of human melanoma spheroids formed under the same conditions was shown to be profoundly affected and marked by upregulation of a number of genes recognized to play a role in melanoma progression [19]. However, it remains unclear whether sphere-formation of human melanoma cells under stem cell conditions, which is often associated with high plasticity, can predict molecular or functional phenotypes associated with aggressiveness. Insights into this possibility could demonstrate the relevance of such a model for patients and clinical transferability.

In this study we evaluated the capacity of human melanoma spheroids formed under stem cell conditions to predict an aggressive phenotype. Melanoma tumor cells are derived from the transformation and proliferation of melanocytes, which arise from the embryonic neural crest. Therefore, we analyzed the molecular and functional phenotypes of melanoma spheroids grown in neural crest cells medium in comparison to their adherent counterparts. Whether derived from metastatic or advanced primary melanoma specimens, these spheroids expressed melanoma-associated markers, displayed high capacity to differentiate along mesenchymal lineages, and showed enhanced expression of the human embryonic stem cells pluripotency markers SOX2, NANOG, KLF4, and/or OCT4, which indicates a higher plasticity. These spheroid cells did not possess the fundamental properties of melanoma initiating cells; they did not show enhanced self-renewal or enhanced tumor formation capacity when compared to their adherent counterparts suggesting that they were not enriched with melanoma initiating cells. However, comparative global gene expression and functional *in vitro* assays indicated that these melanoma spheroid cells exhibit a neural crest cell signature associated with enhanced migratory/invasive and immune evasion capacities, two functions involved in tumor aggressiveness. Thus, human melanoma spheroids in our model showed a more aggressive tumor phenotype and suggested that aggressiveness and heterogeneity of melanoma tumors can be supported by subpopulations other than cancer stem cells. Therefore, they could be suggested as a constructive model to investigate melanoma aggressiveness.

## Materials and Methods

### Cell culture

Melanoma cells were obtained from a lymph node metastasis (SLM8) and from a biopsy of a primary stage IV-cutaneous melanoma (Mela1). Patients signed an informed consent following human ethics committee (Comité consultatif pour la protection des personnes dans les recherches biomédicales, Hôpital Saint Louis, Paris, France), and the study has been approved by the institution. Single-cell suspensions were generated by enzymatic digestion of the biopsy in 2 mg/mL Collagenase (Sigma-Aldrich, Saint-Quentin Fallavier, France) in DMEM/F12 medium (Invitrogen, Cergy Pontoise, France) for 2 h at 37°C. SLM8 and Mela1 cells were grown as monolayer adherent cells in DMEM/F12 supplemented with Penicillin-streptomycin (100 U/mL and 100 µg/mL, Invitrogen), L-Glutamine (2 mM, Invitrogen), and fetal calf serum (FCS, 10%, Invitrogen). Between passages 6 and 12, adherent cultures homogeneously express melanoma markers (HMB-45-reactive antigen, MART1/Melan A, tyrosinase), ensuring their purity from non-tumor contaminating cells. Melanoma multicellular spheroids were generated by culturing these adherent cells after dissociation at a clonal density of 1000 cells/mL in neural crest cells culture conditions, where FCS is replaced by B27 (1×, Invitrogen), EGF (20 ng/mL, Upstate, Millipore, Guyancourt, France), bFGF (20 ng/mL, Invitrogen) and LIF (10 ng/mL, Chemicon International, Millipore) as described [20]. Experiments were performed with individual cells derived from spheroids formed after 11 to 21 days. Spheroids were dissociated as previously described [12]. The expression of frequently tested melanoma markers (HMB-45-reactive antigen, MART1/Melan A, tyrosinase) was verified on adherent cells and on single-cell suspensions from spheroids using anti-pan melanoma (HMB45+M2-7C10+M2-9E3+T311) antibodies cocktail (Novus Biological Inc, Littelton, CO, USA).

### *In vitro* limited dilution assays

*In vitro* self-renewal assessment was performed as described [21]. Briefly, dissociated cells from melanoma adherent and spheroid cultures were serially diluted in appropriate medium and seeded at 1 cell/well. Wells containing spheroids formed from one cell and wells containing one colony of adherent cells were counted after one, two and three weeks.

### Flow Cytometry

Expression of markers was determined by immunostaining as described [22] using: FITC-conjugated anti-αvβ3 (23C6), PerCP-conjugated anti-CD20 (L27), anti-CD146(MCAM) (P1H12), anti-CD80 (L307.4), anti-CD86 (FUN-1) from BD Biosciences (Le Pont de Claix, France); PE-conjugated anti-CD133/2 (293C3) from Miltenyi Biotec (Paris, France), FITC-conjugated anti-ABCG2 (5D3) from (Chemicon International, Millipore); anti-CD166 (L50), anti-nestin (3 k1) from Abcam (Paris, France); anti-MHC I (W6:32), anti-MHC II HLA-DR (L243), HLA-DP (B7/21), HLA-DQ (33.1), anti-CD40 (G28.5) were affinity-purified from ascites using protein A-Sepharose column (Amersham Pharmacia Biotech). Un-conjugated primary antibodies were detected with PE-conjugated goat anti-mouse IgG(H+L) antibody (BD Biosciences). Cells were analyzed using a FACScan flow cytometer (BD Biosciences).

### Immunohistochemistry

Melanoma adherent cells or melanoma spheroid cells were cytocentrifuged onto slides (cytospin). After fixation/permeabilisation with methanol/acetone for 10 min, slides were incubated with anti-pan melanoma (HMB45+M2-7C10+M2-9E3+T311) for 15 min, then with streptavidin alkaline phosphatase for another 15 min. Alkaline phophatase activity was developed using Fuchsin plus Substrate-Chromogen System (DAKOCytomation, Trappes, France) for 10 min.

### Differentiation assays of melanoma cells

Melanoma cells were assessed for their capacity to differentiate along adipogenic and osteogenic lineages. For adipogenic lineage, single-cell suspensions from adherent and spheroid cultures of SLM8 and Mela1 were plated in tissue culture-grade plastic at a density of 50×10^3^ cells/cm^2^ and allowed to differentiate in adipogenic culture medium (PromoCell, Belgium) for two weeks as recommended by the manufacturer. After differentiation, accumulation of lipid vacuoles was visualized by staining with Oil Red O (Sigma-Aldrich). For osteogenic lineage, single-cell suspensions were plated at 3×10^3^ cells/cm^2^ in osteogenic culture medium (PromoCell) for two-three weeks as recommended by the manufacturer. After differentiation, activity of Alkaline Phosphatase was assessed using Alkaline Phosphatase activity detection kit (Sigma-Aldrich) according to the manufacturer's recommendations. Oil Red O-stained cells and cells presenting alkaline phosphatase activity were counted under a light microscope. Assays were performed in triplicates.

### Western Blot analysis

OCT4, NANOG and SOX2 expression was analyzed in total cell extracts (50 µg) separated by 10% SDS-polyacrylamide gel electrophoresis and transferred to nitrocellulose transfer membrane (GE Healthcare Life Sciences, France). The membranes were washed in Tris-buffered saline with Tween (10 mM Tris-HCl, pH 8, 150 mM NaCl, and 0.05% Tween 20), blocked 1 h at room temperature with 5% nonfat milk or 1% BSA in Tris-buffered saline with Tween, then probed 1 h at room temperature with anti-OCT4 (Abcam,), anti-NANOG (Abcam), and anti-SOX2 (Santa Cruz Biotechnologies, Germany). After incubation with horseradish peroxide-conjugated secondary antibody, immunoreactive proteins were detected by ECL detection system (GE Healthcare). Protein concentrations were determined by using the BCA protein assay kit (ThermoScientific Biosciences, France).

### Migration and invasion assays

Migration and invasion assays were performed in 24-well plates transwell chambers with 8 µm-pore polycarbonate filter inserts (BD Biosciences). 5×10^4^ dissociated spheroid or adherent cells were seeded on uncoated inserts whereas 10^5^ cells were seeded on collagen type I - or Matrigel-coated inserts in 100 µL of serum-free medium. The lower chambers were filled with 0.5 ml of 10% FCS-supplemented DMEM/F12 medium. After 24 h and/or 48 h, cells on the upper side of the filter were removed and the cells on the lower surface of the insert were fixed and stained with Diff-Quik staining Kit (Medion Diagnostics; Düdingen, Switzerland). The number of stained cells was counted under a light microscope. Assays were performed in triplicates.

### Immunogenicity assays

Peripheral blood mononuclear cells (PBMCs) were prepared by centrifugation on a Ficoll-Hypaque density gradient (Sigma-Aldrich) of blood samples from healthy donors characterized for their MHC haplotype. The haplotypes of SLM8 and Mela1 melanoma cells were HLA-DRB1*07/11, HLA-DQB1*02/03, and HLA-DRB1*13/07,HLA-DQB1*06/02, respectively. To determine the capacity of melanoma cells to elicit an allogeneic response, 1×10^5^ responding PBMCs were co-cultured with either allogeneic stimulatory PBMCs (1×10^5^ cells) or dissociated melanoma spheroid cells or melanoma adherent cells (1×10^4^ cells) treated with mitomycin C (20 and 50 µg/mL, respectively, for 30 min) in U-bottom 96-wells plates. PBMCs proliferation was determined by ^3^H-thymidine pulse (1 µCi/well) for 18 h prior to the end of experiment. In assays determining melanoma suppressive effect, dissociated spheroid cells or melanoma adherent cells treated with mitomycin C (50 µg/mL) were co-cultured with HLA-matched PBMCs labeled with CFSE (1 µM for 10 min) in the presence of phytohemagglutinin (PHA). At the indicated time point, cells were stained with eFluor780-conjugated anti-CD3 (UCHT1, eBiosciences), PE-conjugated anti-CD25 and 7AAD (BD Biosciences) to determine T cell activation, proliferation and death. Cells were then analyzed by Canto II flow cytometer (BD Biosciences).

### RNA Isolation and Reverse Transcription–PCR

Total RNA extraction, the conditions of reverse transcription, and the quantitative PCR (Q-PCR) and semi-quantitative PCR (sq-PCR) analysis were carried out as we described in [22]. For Q-PCR, specificity of PCR amplicons was confirmed by melting curve analysis and relative expression of tested genes was calculated using the target threshold (C_T_) value and the 2^−ΔΔCt^ method [23]. For each gene, values were averaged over three independent measurements and relative transcript level was calculated. For sq-PCR, the number of cycles was evaluated for each primer set and experiment as maintaining an exponential amplification. For both Q- and sq-PCR, RPL13 was used as the housekeeping gene, and the nucleotide sequences of the different primers along with the corresponding annealing temperature and number of cycles performed are indicated in tables S10 and S11 (supplementary information).

### Affymetrix Exon Array hybridization

One µg of total RNA from SLM8 and Mela1 adherent and spheroid cells were extracted using Trizol (Sigma-Aldrich) then labeled with reagents from Affymetrix. Hybridization cocktails containing 5–5.5 µg of fragmented, end-labeled single-stranded cDNA were prepared and hybridized to Affymetrix-GeneChip® Human Exon 1.0 ST arrays (Affymetrix). Processed arrays were scanned using the GeneChip Scanner 3000 7G. Affymetrix Expression Console Software was used to perform quality assessment.

#### Data analysis

Data were processed by the EASANA® analysis system and visualization interface (GenoSplice technology, Paris, France), which is based on the FAST DB annotations [24]. Array data were normalized using quantile normalization method and corrected using the antigenomic probes [25], [26], [27], [28], [29]. Probe selection was made as described [26],[27],[28],[29]. Only probes targeting exons annotated from full-length cDNA were kept for analysis. Among these pre-selected probes, bad quality probes and probes with intensity signal too low compared to antigenomic background probes with the same GC content were disregarded. Only probes with a DABG p-value≤0.05 in at least half of chips were considered for further statistical analysis as described [26], [27], [28], [29]. The experiment (from cell injection to array hybridization) was performed three times. Paired statistical analyses were performed using Student's paired *t*-test on the gene signal intensities (gene level) as described [26], [27], [28], [29]. Results were considered statistically significant at *p*-values≤0.05 and fold-change ≥1.5. Hierarchical clustering was carried out to cluster among the gene signal intensities and among the samples with Mev4.0 software from TIGR (The Institute of Genome Research). Euclidean distance with complete linkage was used. The functional analyses were generated through PANTHER [30].

### Statistical analysis

Results are presented as mean values ± SD of at least three independent experiments. Statistical differences between samples were determined using OneWay ANOVA followed by Student-Newman-Keuls method (SigmaStat software) and a *p*-value of <0.05 was considered significant.

## Results

### Melanoma cells expanded as spheroid cultures in neural crest cell medium show high plasticity and enhanced expression of human embryonic stem cells pluripotency transcription factors

**Figure 1:**
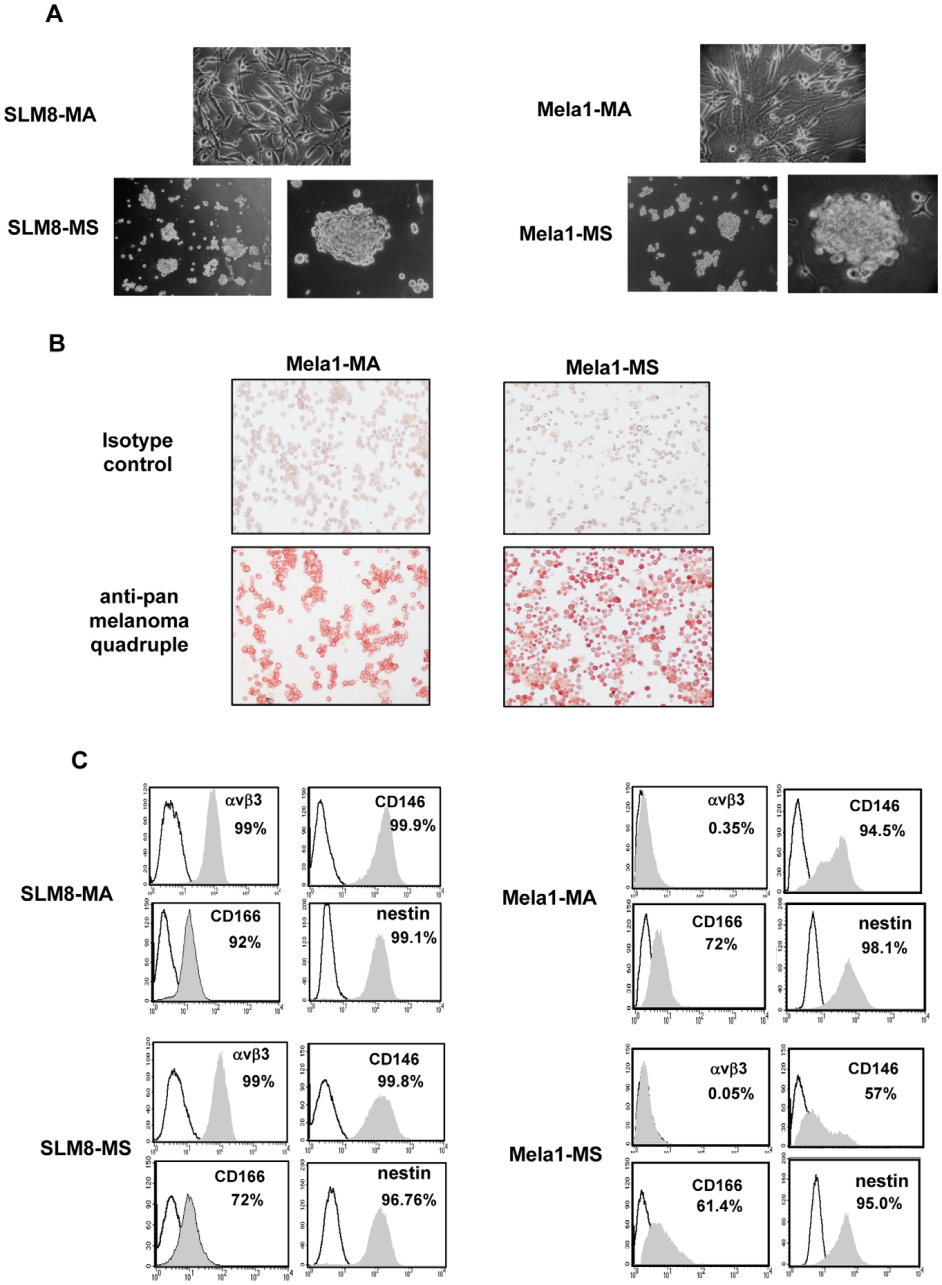
Melanoma spheroid cells grown under neural crest cells conditions and their adherent counterparts have common phenotypes. **A**. SLM8 and Mela1 monolayer adherent melanoma cells (SLM8-MA and Mela1-MA) form multicellular spheroids (SLM8-MS and Mela1-MS) under neural crest cell conditions (magnification ×50 for MA cells; ×20 and ×50 for MS cells). **B**. Mela1-MS, similarly to Mela1-MA, cells express melanoma markers, as determined by immunostaining with anti-pan melanoma antibodies (HMB45+M2-7C10+M2-9E3+T311) and Mayer's hematoxylin counterstaining. **C**. Expression of αvβ3, CD146, CD166 and nestin was determined by flow cytometry (grey filled histogram versus unfilled histograms for respective isotype control).

Whether from a lymph node metastasis (SLM8) or from a primary stage IV-cutaneous lesion (Mela1), melanoma cells when seeded at a clonal density in neural crest cells medium form small floating spheroid structures (15–30 cells) within 2 days of culture that grow to approximately 200 cells each within 2 weeks (Fig. 1A). Similar to their adherent counterparts, both Mela1 (Fig. 1B) and SLM8 (data not shown) spheroid cells stain positively for at least one of the frequently tested melanoma markers HMB-45, MART1/Melan A and/or tyrosinase (Fig. 1B). Spheroid cells derived from SLM8 expressed melanoma-associated markers integrin αvβ3 [31] and CD146 (M-CAM) [32], the neuroectodermal stem cells marker nestin [33], [34], and the mesenchymal stem cells surface molecule CD166 [34], [35] (Fig. 1C). Mela1 spheroid cells also expressed CD146, CD166, nestin, but not integrin αvβ3 (Fig. 1C), which could be due to the tumor progression state of Mela1 cells given that αvβ3 is reported as mainly expressed in metastatic regions [31]. Spheroids showed an expression profile similar to their adherent counterparts, albeit for decreased expression of melanoma-associated marker CD146 (Fig. 1C).

**Figure 2:**
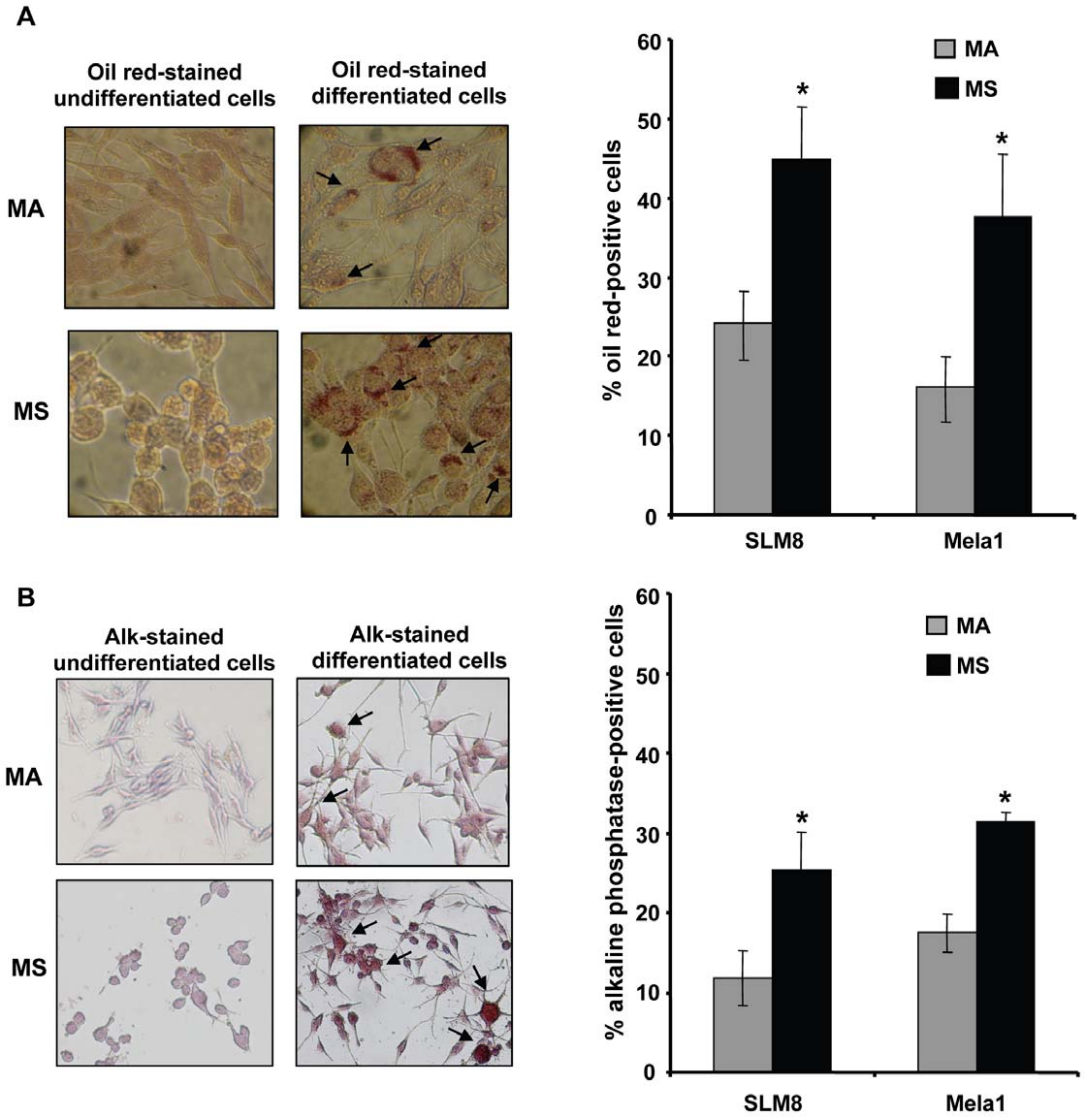
Melanoma spheroid cells display higher capacity to differentiate along mesenchymal lineages. **A**. Adipogenic differentiation of melanoma adherent (MA) cells and spheroid (MS) cells was assessed by Oil Red O staining. Left panel shows Mela1 undifferentiated and differentiated MA and MS cells (magnification ×100). Right panel demonstrate the extent of adipogenic differentiation of Mela1 and SLM8 melanoma cells as percentage of Oil Red O stained cells. **B**. Osteogenic differentiation of MA and MS cells was assessed by alkaline phosphatase (AP) activity detection. Left panel shows SLM8 undifferentiated and differentiated MA and MS cells (magnification ×10). Right panel presents the percentages of cells presenting AP activity. Results are mean percentage values of cell counts obtained in 15 independent microscope fields ± SD, *p<0.001.

Given the neuroectodermal origin of melanocytes, we evaluated the capacity of these spheroid cells to differentiate along the mesenchymal lineages. We compared the extent of adipogenic and osteogenic differentiation of SLM8 and Mela1 spheroid cells *versus* their adherent counterparts. Under conditions sustaining adipogenic differentiation, both spheroids and adherent cells from Mela1 (Fig. 2A left panel) and SLM8 (Fig S1 A, Supplementary data) presented Oil Red O-stained lipid vacuoles. Twenty-four ±5.17% and 16.2±5.4% of SLM8 and Mela1 adherent cells, respectively, were Oil Red O-positive compared to 44.9±7.3% and 37.6±8.6% of SLM8 and Mela1 spheroids, respectively (Fig. 2A right panel). SLM8 (Fig. 2B left panel) and Mela1 (Fig S1 B, supplementary data) spheroid cells and their adherent counterparts were also able to differentiate along osteogenic lineage in osteogenic medium. Nearly 25.4±4.7% and 31.5±1.0% of SLM8 and Mela1 spheroids and 11.9±3.4% and 17.4±2.3 of their adherent counterparts, respectively, presented alkaline phosphatase activity (Fig. 2B right panel). These results show higher capacity of spheroid cells to differentiate along mesenchymal lineages and suggest that melanoma spheroids grown under neural crest cell conditions are enriched with multipotent cells when compared to their adherent counterparts.

**Figure 3:**
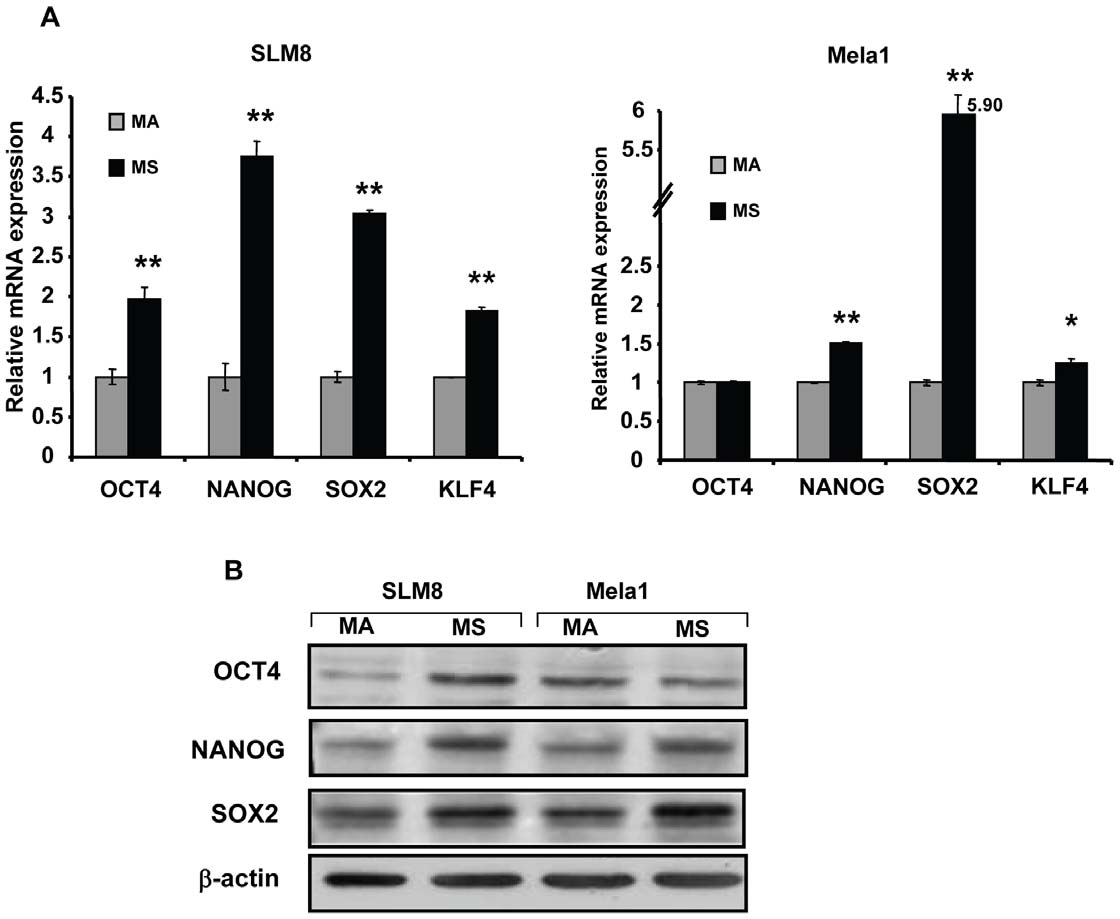
Melanoma spheroid cells display higher expression of pluripotency transcription factors. **A**. Expression of pluripotency transcription factors OCT4, NANOG, SOX2 and KLF4 by spheroid (MS) and adherent (MA) cells from SLM8 and Mela1 was determined by quantitative PCR. Results are presented as mean values of relative mRNA expression ± SD from three independent experiments; *p<0.05; **p<0.005. **B**. Western blots with specific antibodies demonstrating the up-regulation of OCT4, NANOG and SOX2 expression in MS cells compared to MA. Re-blotting with anti-beta-actin ensured equal loading.

As OCT4, NANOG, SOX2, and KLF4 are recognized as regulators of cells pluripotency and as human embryonic stem cells markers [36], [37], [38], [39], we investigated the expression of these transcription factors in our model. Quantitative PCR analysis showed that spheroid cells from SLM8 and Mela1 express significantly higher levels of NANOG, SOX2 and KLF4 compared to their adherent counterparts (Fig. 3A). Spheroid cells from SLM8, but not from Mela1, also showed enhanced expression of OCT4 (Fig. 3A). Western blot analysis also showed that SLM8 spheroid cells express enhanced levels of OCT4, NANOG and SOX2 over their adherent counterparts (Fig. 3B). Compared to their adherent counterparts, Mela1 spheroid cells displayed higher levels of SOX2 and NANOG, but not OCT4 (Fig. 3B). These results show that melanoma spheroid cells grown under neural crest cell conditions display high plasticity as they are enriched with multipotent cells and express higher levels of pluripotency regulators. This supports the notion that these melanoma spheroids could be enriched with cells possessing stem cell-like properties.

### Melanoma spheroid cells do not display the properties of melanoma stem cell-like or initiating cells

**Figure 4:**
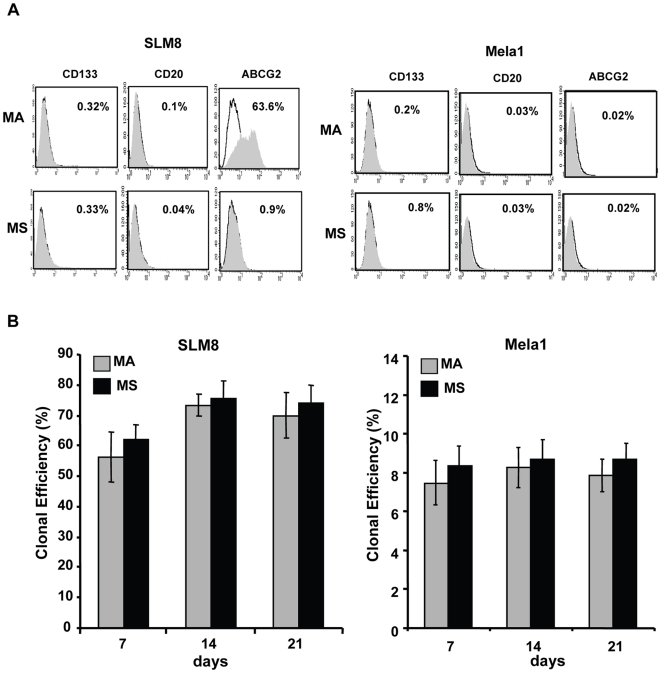
Melanoma spheroids are not enriched with putative melanoma CSCs. **A**. Expression of CD133, CD20 and ABCG2 by SLM8- and Mela1-MA and -MS cells determined by flow cytometry (grey filled histogram versus unfilled histograms for respective isotype control). **B**. Cloning efficiency of spheroid (MS) and adherent (MA) cells from SLM8 and Mela1 as determined by limiting dilution assays over three weeks. Results are expressed as mean percentage values ± SD from 4 different experiments.

We then investigated if spheroids are enriched with melanoma stem cell-like or initiating cells. We first examined the expression of putative markers of these cells CD20, CD133, ABCG2 and ABCB5 [12], [13], [40]. Flow cytometry showed that melanoma spheroid cells whether derived from SLM8 or from Mela1 were negative for CD133, CD20 and ABCG2 (Fig. 4A). Indeed, melanoma spheroid cells displayed the same expression pattern as their adherent counterparts with the exception of ABCG2, which was expressed by 63.6% of SLM8 adherent cells. The expression of ABCB5 was not detectable by immunostaining, western blot, or flow cytometry analysis in either spheroid or adherent cells from SLM8 and Mela1 melanoma (Fig S3, Supplementary data). The expression of ABCG2 or ABCB5 is often associated with cells having the property of side-population cells [13], which usually presents cells having drug-efflux activity [41], [42]. Therefore, we conducted drug efflux experiments and found that neither adherent nor spheroid cells from both SLM8 and Mela1 have the property of side-population cells (Fig S2, Supplementary data) thus, supporting the above results. The melanoma stem cell-like or initiating cells marker expression does not seem to be related to culture duration given that phenotype analysis at different time points during culture yielded similar results (data not shown).

We then investigated through limited dilution assays the self-renewal potential of melanoma spheroid cells in comparison with their adherent counterparts. SLM8 and Mela1 spheroids were dissociated and plated into 96 wells as a single cell per well, and the number of wells containing a single cell able to generate spheroid structures was determined. Single cells derived from SLM8 and Mela1 spheroids generated spheroids at 60–70% and 8–9%, respectively (Fig. 4B), showing the potential of these clonal tumor spheroids to generate colonies although with different efficiency. Interestingly, adherent SLM8 and Mela1 cells demonstrated clonal efficiency similar to their respective spheroid counterparts when they were seeded at 1 cell/well. These results indicate that spheroid cells in our model do not show enhanced self-renewal when compared to their adherent counterparts. Furthermore, these melanoma spheroid cells displayed the same capacity of tumor formation as their adherent counterparts when serially xenotransplanted into nude mice (Fig S4, Supplementary data). Together these results indicate that spheroids are highly plastic cells but are not enriched with melanoma stem cell-like or initiating cells given that they do not present enhanced tumorigenic and self-renewal capacities when compared with their adherent counterparts.

### Gene expression profile of melanoma spheroid cells versus their adherent counterparts

To determine whether melanoma spheroids in our model could predict a molecular or functional phenotype, we performed gene expression profiling experiments using Affymetrix microarrays. The data have been deposited in NCBI's Gene Expression Omnibus and are accessible through GEO Series accession number GSE26980 (http://www.ncbi.nlm.nih.gov/geo/query/acc.cgi?acc=GSE26980).

**Figure 5:**
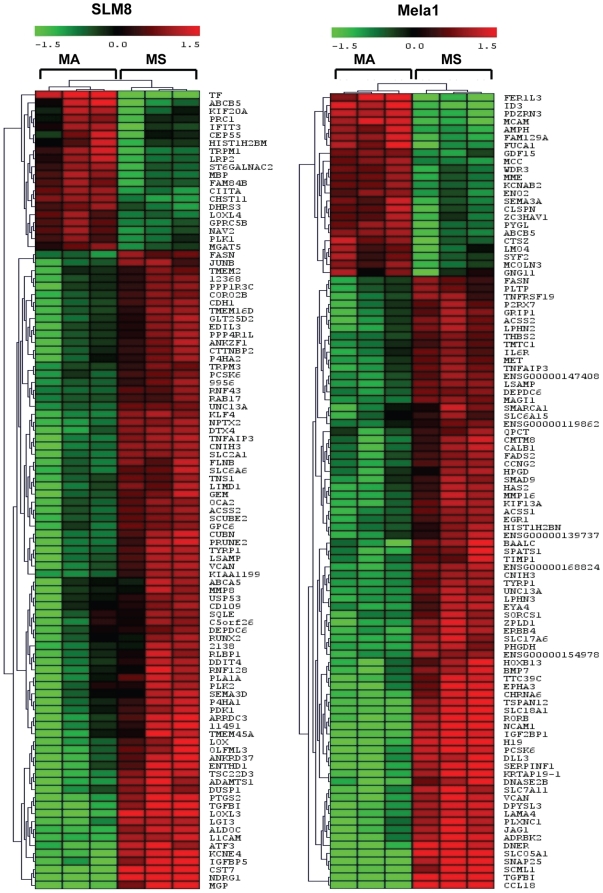
Global gene expression profiling. Affymetrix microarray was performed with mRNA extracted from SLM8- and Mela1-spheroid (MS) and -adherent (MA) cells. Heatmaps of the 100 more regulated genes for SLM8 and Mela1 cells.

Global probing for differences in gene expression in spheroid cells compared to their adherent counterparts showed statistically significant altered expression of 273 (up) and 189 (down) genes for SLM8, and 185 (up) and 205 (down) genes for Mela1 in the microarray using a paired *t*-test with *p*-values≤0.05, fold-change ≥1.5 and excluding non-annotated genes (Fig S5, Supplementary data). Heatmaps were established with the 100 more regulated genes for both SLM8 and Mela1 cells (Fig. 5). Changes in gene expression were found to be common but also distinct for each cell line: among all the regulated genes, 58 genes were found to be commonly up-regulated in Mela1 and SLM8 spheroid cells and 7 to be commonly down-regulated (Fig S5 and Table S1, Supplementary data).

**Figure 6:**
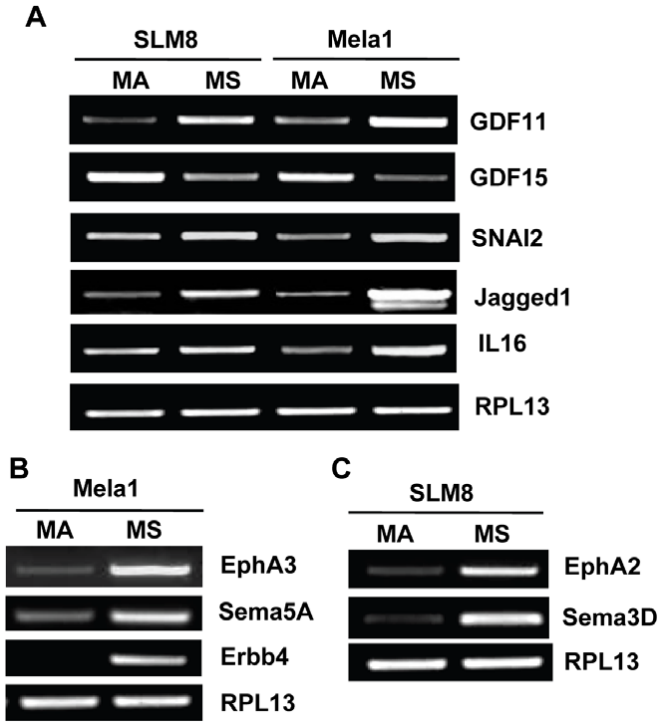
Differential expression of representative genes. RT-PCR of representative genes was performed with mRNA extracted from SLM8- and Mela1-spheroid (MS) and -adherent (MA) cells, using specific primers as indicated in Table S10 (supplementary data). RPL13 is used as housekeeping gene to ensure equal loading.

RT-PCR was employed to confirm the differential expression of 10 representative genes, which were chosen based on their implication in neural crest cells function, particularly adhesion and motility, in tumor progression and/or regulation of immune response. A good correlation between microarray and RT-PCR data was observed for most of the validated genes (Fig. 6). SLM8 and Mela1 spheroid cells expressed enhanced levels of GDF11 while showing decreased expression of GDF15; both genes belonging to the BMP/GDF group of the TGFβ superfamily and being regulators of cell growth and differentiation in embryonic and adult tissues and in tumors progression [43]. Both spheres populations also over-expressed JAG1 (Jagged 1), a ligand of Notch receptor that is involved in developmental processes as well as in the development of regulatory T cells in both human and mouse systems [44], [45], [46]. Even though at different levels, Mela1 and SLM8 spheroid cells also showed upregulated expression of IL-16, a pleiotropic cytokine that is a natural ligand of CD4 and is a chemo-attractant for CD4^+^ T cells, particularly T regulatory cells [47], and SNAI2 (Slug), a master regulator of neural crest cell specification and migration and is implicated in melanoma progression [48], [49] (Fig. 6A). Mela1 spheroid cells over-expressed EphA3, Sema5a and ErbB4 (Fig. 6B) whereas SLM8 spheroid cells over-expressed EphA2 and Sema3D (Fig. 6C). While not always identical, these over-expressed genes by melanoma spheroid cells grown under neural crest cells conditions do belong to the same families and/or participate in similar processes such as embryonic neural crest cell migration and cell fate regulation [43],[48], tumor progression [50], and growing evidence also indicates their involvement in regulation of immune response [51], [52].

### Melanoma spheroid cells transcription signature puts them forward as highly plastic cells that could assume the role of aggressive melanoma cells

**Figure 7:**
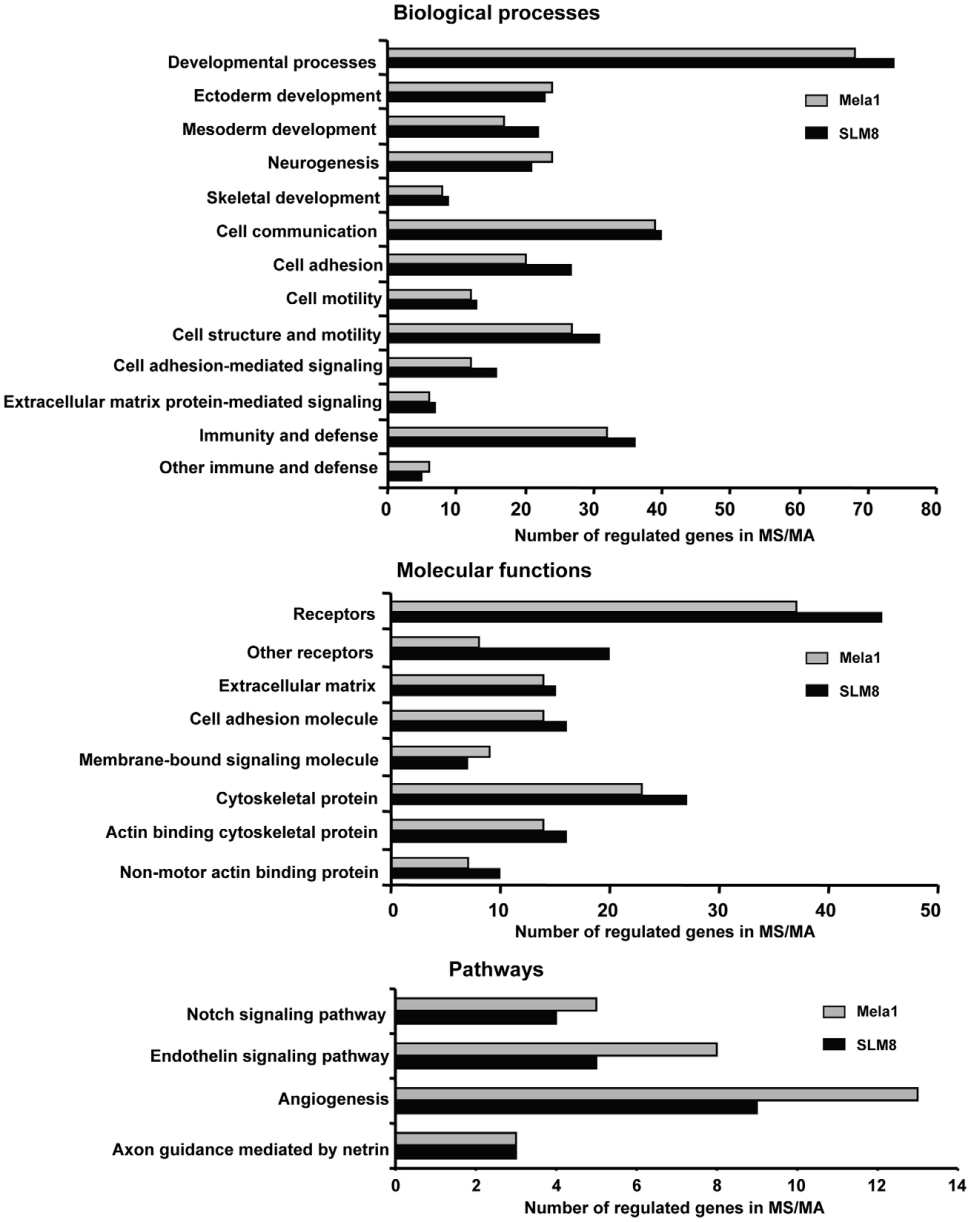
Global functional analysis of modulated genes. PANTHER software was used to determine the regulated biological processes (upper panel), the molecular functions (middle panel), and the signaling pathways (lower panel). Bars represent the number of regulated genes.

Using the Panther software, we then defined the biological processes, molecular functions and signaling pathways involving the regulated genes in spheroid cells compared to adherent cells (Fig. 7). The biological processes profiling revealed that for both SLM8 and Mela1 cells, the highest number of regulated genes in spheroid cells concerned developmental processes (Fig. 7 and Table S2, supplementary data), which encompass ectoderm, mesoderm and skeletal development, and neurogenesis (Fig. 7 and Table S3, supplementary data). This suggests gene expression changes in melanoma spheroids in agreement with their higher expression of pluripotency regulators and differentiation capacity. Besides, various genes involved in cell structure, motility, and adhesion, as well as extra-cellular matrix protein-mediated and cell adhesion signaling were differentially expressed (Fig. 7 and Table S4 and S5 supplementary data) and were suggestive of an enhanced capacity of the spheroid cells to migrate. Interestingly, both Mela1 and SLM8 spheroid cells also regulated several genes involved in immunity and defense and/or immune-related processes (Fig. 7 and Table S6 supplementary data).

In agreement, the molecular profiling revealed in spheroid cells a high number of regulated genes encoding for cytoskeleton, extra-cellular matrix, cell adhesion, immune response-related receptors and membrane bound signaling proteins (Fig. 7 and Table S7 and S8, supplementary data). Finally, the pathway profiling revealed that genes implicated in Notch, Endothelin, angiogenesis as well as axon guidance pathways are also differentially regulated in spheroid cells (Fig. 7 and Table S9 supplementary data), which could be either directly or indirectly linked to the above underscored biological processes. Besides concurring with higher plasticity, the global functional analysis of melanoma spheroid cells transcriptional profile also suggested that a migratory/invasive and/or immune-function modulating program could be associated with these melanoma spheroid cells.

### Melanoma spheroid cells display higher *in vitro* migratory/invasive capacity

**Figure 8:**
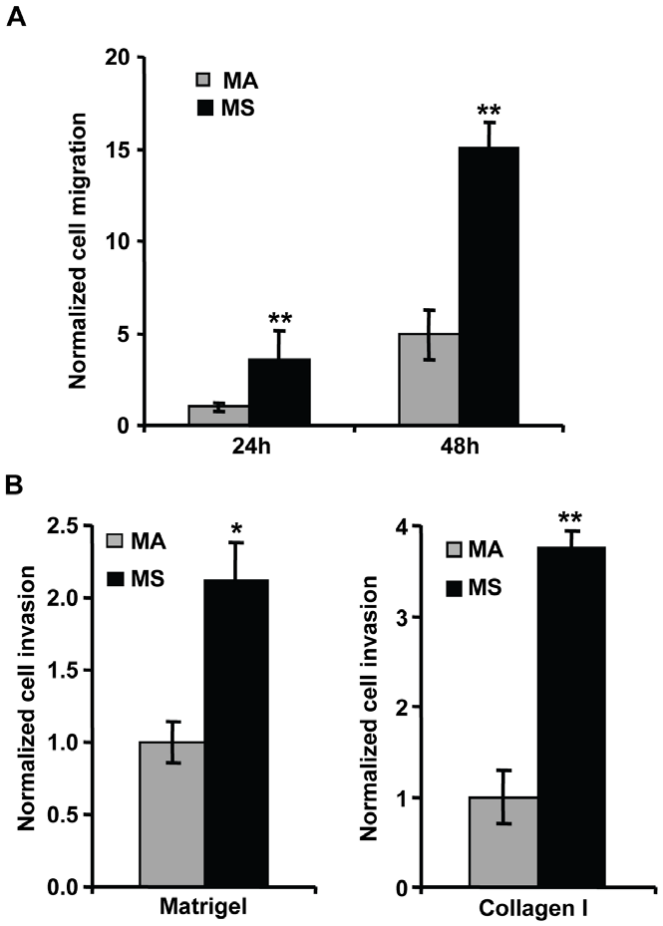
Melanoma spheroid cells have enhanced migratory/invasive capacities. **A**. Chemotaxis (uncoated inserts) and **B**. invasion (matrigel- and collagen I-coated inserts) capacities of SLM8 adherent (MA) and spheroid (MS) cells were assessed using Transwell migration chambers. Experiments were performed three times in duplicate and results are presented as relative values against the number of adherent cells that have migrated (24 h and 48 h) or invaded at 48 h. Bars: SD, *p<0.05; **p<0.005.

To corroborate the obtained melanoma spheroid cells transcriptional profile we conducted *in vitro* assays to evaluate the migratory/invasive capacity of melanoma spheroid cells in comparison with their adherent counterparts. Using Transwell migration chambers, we found that spheres grown under neural crest cells conditions display a significant increase in cell motility. Compared to SLM8 adherent cells, SLM8 dissociated spheroid cells showed a 3.6-fold (p<0.05) and 3-fold (p<0.005) increase in chemotactic potential at 24 and 48 h, respectively (Fig. 8A). These cells also demonstrated higher capacity to invade both Matrigel- and collagen I-coated inserts in Transwell migration chambers. Significantly higher numbers of SLM8 spheroid cells were able to invade Matrigel and collagen I as compared to SLM8-adherent cells (p<0.05 and p<0.005, respectively) (Fig. 8B). Similar results were also obtained when spheroid and adherent cells from Mela1 were compared for their migration and invasion capacities (data not shown). These results indicate that melanoma spheroid cells, compared to their adherent counterparts are endowed with higher migratory/invasive capacity attributing to spheroid growth pattern a functional phenotype associated with tumor aggressiveness.

### Melanoma spheroid cells are more efficient in modulating the immune response

**Figure 9:**
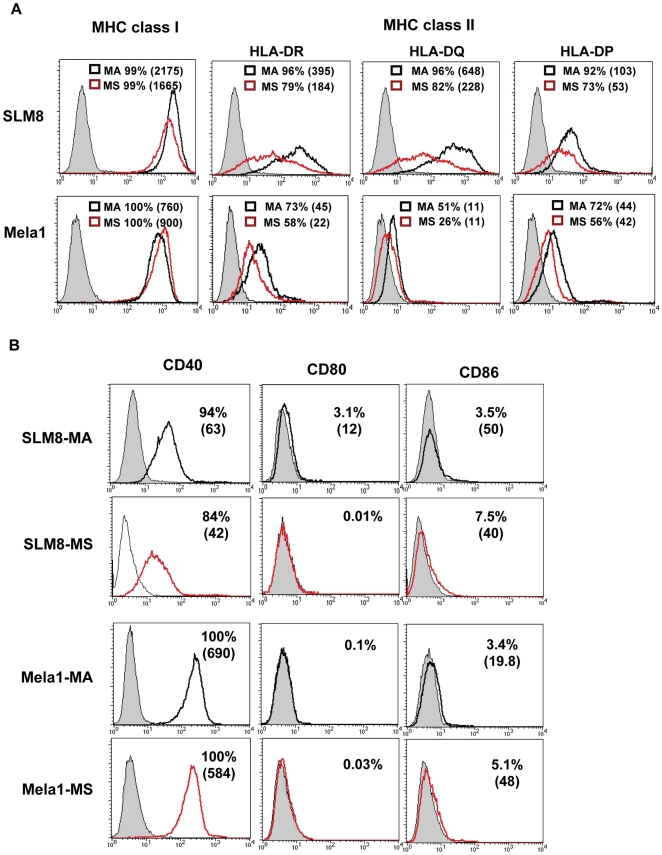
Expression of immunological relevant molecules by melanoma spheroid and adherent cells. Expression of MHC I and MHC II (HLA-DR, HLA-DQ and HLA-DP) molecules (**A**) and co-stimulatory CD40, CD80, and CD86 molecules (**B**) was determined by flow cytometry by SLM8 and Mela1 adherent (MA) and spheroid (MS) cells. Unfilled red histograms for spheroids or unfilled black histograms for adherent cells versus grey filled histograms for respective isotype control. Percentages of positive cells and the mean fluorescence intensity () are indicated.

We then tested whether an immune-function modulating program could be associated with the melanoma spheroid cells. First, we evaluated the expression of immunologically relevant cell surface molecules by SLM8 and Mela1 dissociated spheroid and adherent cells (Fig. 9A and 9B). SLM8 adherent cells were positive for MHC class I, MHC class II (HLA-DR, DQ, and DP), CD40 but very weakly positive for co-stimulatory molecules CD80 (B7.1) and CD86 (B7.2). Mela1 adherent cells were similarly positive for MHC class I, CD40, expressed lower levels of MHC class II (HLA-DR, DQ, and DP), and were negative for CD80 and weakly positive for CD86. SLM8 and Mela1 spheroid cells displayed comparable immunological phenotypes to their adherent counterparts although with a decrease in the expression of MHC class II molecules.

**Figure 10:**
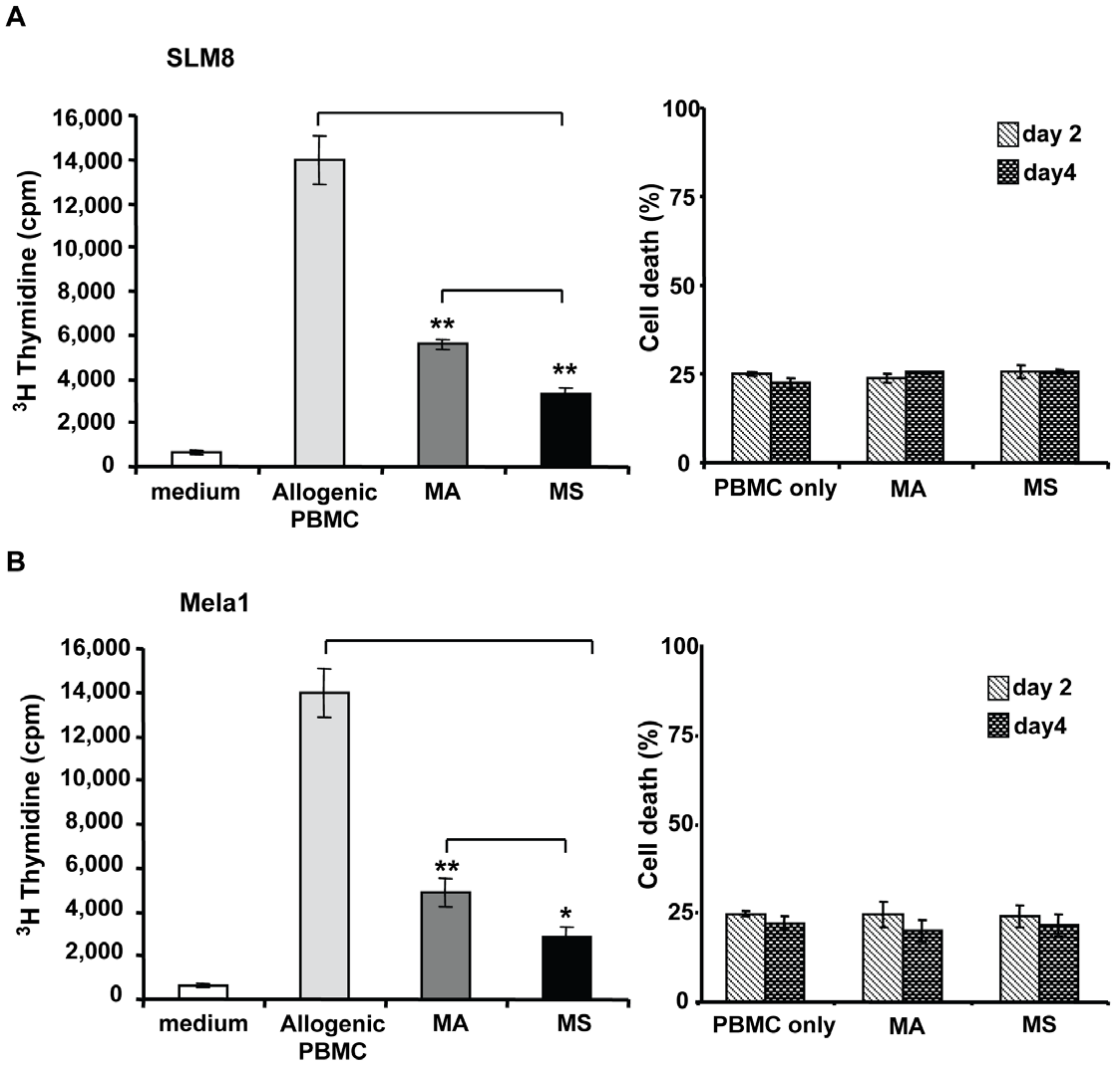
Melanoma spheroid cells elicit lower allogeneic response. Unfractionated PBMCs were cultured in microtiter wells with medium alone, allogeneic PBMCs, melanoma adherent cells or melanoma spheroid cells from either SLM8 (**A**) or Mela1 (**B**). PBMC proliferation was evaluated at day 6 by ^3^H-Thymidine incorporation (left panel) and cell death was evaluated at days 2 and 4 by 7-AAD staining and flow cytometry analysis (right panel). Experiments were performed in quadruplicate and results are presented as mean values ± SD from 4 independent experiments (*p<0.05; **p<0.001).

The allogeneic mixed lymphocyte reaction (MLR), where lymphocytes from MHC incompatible individuals will stimulate each other to proliferate significantly, has served as an important experimental system for elucidating the cellular and molecular basis of human lymphocyte responses. The one-way MLR test where the lymphocytes from one of the individuals are inactivated by mitomycin C or irradiation, thereby allowing only the untreated remaining population of cells to proliferate in response to foreign MHC antigens, has been adapted for elucidating the immunological properties of various cells including mesenchymal stem cells [53]. Therefore, to provide insights into the immunological properties of melanoma spheroid cells, we sought to investigate their capacity to elicit an allogenic response in a one-way MLR. Un-fractionated allogeneic peripheral blood mononuclear cells (PBMCs) were co-cultured with mitomycin-treated cells from melanoma spheroids or adherent cultures in 96 wells for 6 days. Un-fractionated PBMCs responded vigorously to mitomycin-treated allogenic PBMCs (Fig. 10). The proliferative response to SLM8 and Mela1 adherent cells from the same donor was 60% and 66% lower, respectively, whereas that of SLM8 and Mela1 spheroid cells was 77% and 79% lower, respectively (Fig. 10A and 10B). Co-culturing with higher titers of melanoma cells did not enhance the immunogenicity of either spheroid or adherent cells (data not shown). To exclude apoptotic cell death as a potential cause of less efficient proliferation, we quantified cell death at days 2 and 4 of the allogenic co-cultures by 7-AAD staining and flow cytometry. We found that neither melanoma adherent nor spheroid cells from both SLM8 and Mela1 induced cell death in PBMCs above baseline levels. Although SLM8 cells express higher levels of MHC class II molecules than Mela1 cells, both cell lines elicited a comparable allogeneic response, which is probably due to the MHC haplotypes which also control the allogeneic response and which are different between SLM8 and Mela1. These results indicate that although both melanoma cell populations have poor stimulatory function, melanoma spheroid cells are significantly less efficient than their adherent counterparts in eliciting an allogeneic response.

**Figure 11:**
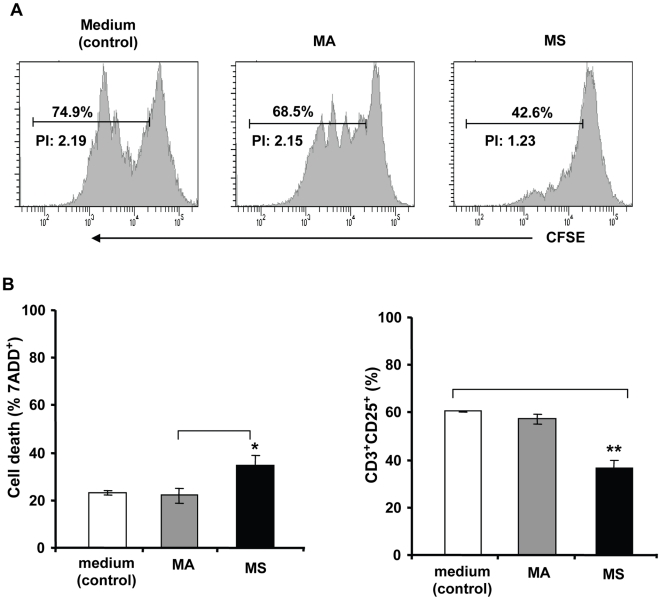
SLM8 spheroid cells inhibit PHA-induced T cell activation and proliferation. PBMCs from MHC-matched healthy donors (n = 3) stimulated with PHA in the presence or absence of SLM8 spheroid (MS) or their adherent (MA) counterparts. **A**. Inhibition of T cell proliferation as determined by FACS analysis of T-cell CFSE labeling as described under material and methods. Percentages of dividing T cells and the mean number of divisions of the responding cells, proliferation index (PI) are indicated. **B**. Analysis of T cell death by 7-AAD staining (left panel) and expression of CD25 as a marker of T cells activation (right panel) by flow cytometry. Experiments were performed in duplicate and results are presented as mean values ± SD from 3 independent experiments (*p<0.05; **p<0.001).

We then sought to determine the effects of melanoma spheroid and adherent cells on mitogen-activated T cell proliferation. PBMCs from healthy donors (n = 3) having an MHC haplotype compatible with SLM8 (SLM8-matched) were labeled with CFSE and then activated with phytohemagglutinin (PHA) in the presence or absence of SLM8 adherent or spheroid cells for 4 days. T cell proliferation was then assessed by FACS analysis of T-cell CFSE labeling. As shown in figure 11A, the presence of SLM8 adherent cells decreased the PHA-induced T cells proliferation only by 8.5% whereas the presence of SLM8 spheroid cells induced a reduction of 43%. Analysis of cell death and expression of CD25 as a marker of T cell activation showed that in the presence of SLM8 spheroid cells, but not adherent cells, T cell death increased from 23% (baseline) to 34.5% while the percentage of CD3^+^CD25^+^ cells decreased from 60.5% (baseline) to 36.6% (Fig. 11B). These results suggest that although adherent melanoma cells are able to inhibit T cell proliferation, melanoma spheroid cells are endowed with higher capacity to inhibit both T cells activation and proliferation. Together our findings attribute a novel immune-modulator function to highly plastic melanoma spheroid cells and suggest specific roles for these spheroids in the evasion of antitumor immunity.

## Discussion

Melanoma tumors are recognized for their heterogeneity and plasticity, which challenges the development of efficient therapies. In this study we showed that melanoma cells expanded as spheroid cultures under neural crest cells conditions have a neural crest cells transcriptional signature associated with high transdifferentiation capacity. Importantly, these spheroids are endowed with enhanced migration/invasion capacities and more efficient immune-modulator function, but are not prominently enriched with melanoma stem cell-like or initiating cells. Our study reveals a previously unrecognized aspect of tumor cells expanded as spheroid cultures by attributing a novel immune-modulator function to more plastic and invasive cells. Thus, melanoma spheroid growth in our conditions predicted a more aggressive tumor phenotype while corroborating its limited efficiency to enrich or identify cancer stem or tumor initiating melanoma cells.

It has been proposed that the aggressiveness and heterogeneity of tumors is supported by rare cell subpopulations within tumor; the cancer stem cells (CSCs), also known as tumor initiating cells. The notion of CSCs and the definition of CSC populations are not yet fully clear in melanoma [54], [55], [56]. Although debated, the CSC notion has been supported by several studies showing prospective purification of melanoma cell subpopulations that express different markers and possess the fundamental CSC properties of enhanced self-renewal, differentiation and enhanced tumorigenic capacity [12], [13], [40], [57], [58]. Expanding tumor cells as spheroid cultures has been used by several groups to enrich and identify CSCs or tumor initiating cells [12], [13], [14], but its utility in identifying melanoma-initiating cells has been recently challenge [15]. Melanoma spheroids in our model presented higher transdifferentiation capacity, which is in accord with previous studies [12], [13]. However, in contrast to [12], [13], [14] our spheroid cells did not show higher tumorigenic or extensive self-renewal capacities when compared to their adherent counterparts. This indicates that both adherent and spheroid cells in our model contain tumor cell subpopulations with capacity to form tumors but melanoma spheroids are not prominently enriched with tumor initiating cells. This is supported by our data showing no increase in the expression of markers, such as CD20 and CD133, previously associated with melanoma stem cell-like or initiating cells, in melanoma spheroids when compared to their adherent counterparts [12], [13]. Our global gene analysis showed that the melanoma initiating cells marker ABCB5 is downregulated in both SLM8 and Mela1 spheroids compared to their adherent counterparts, although we did not detect ABCB5 at the protein level nor find side-population cells described to be associated with ABCB5 expression [59] in either population. Our results are in line with the limited efficiency of sphere-formation assay as a surrogate tool for enrichment and/or identification of CSCs in melanoma [15]. In addition, our data suggest that aggressiveness and heterogeneity of melanoma tumors may be supported by subpopulations other than CSCs.

Spheroid cells in our model displayed a transcriptional profile marked by the regulation of genes involved in several developmental processes, increased expression of human embryonic stem cells pluripotency markers, and enhanced transdifferentiation capacity. Spheroid cells prominently expressed the pluripotency factors NANOG, KLF4, SOX2 and OCT4, although differences in the expression levels were observed between Mela1 and SLM8. These factors were shown to reprogram terminally differentiated cells to generate induced-pluripotent cells similar to embryonic stem cells [36], [37], [39]. Interestingly, global gene expression analysis also showed that even though the genes modulated in Mela1 and SLM8 spheroid cells are different, the affected developmental processes in both spheroid cells are overall similar, in particular the neural crest cell process. Our results suggest that the spheroids grown under neural crest cells conditions could represent a cellular population that is enriched with melanoma cells having enhanced neural crest cell plasticity; given their transcriptional signature, their higher expression of pluripotency regulators, and their enhanced adipogenic and osteogenic differentiation. Melanoma cells injected in chick's embryo were shown to reverse and behave as neural crest cells suggesting, the presence, within the bulk of the tumor, of melanoma cells that can acquire the capacity to reverse to neural crest cell state [60]. In our model, melanoma spheroids express HMB-45-reactive antigen, MART1/Melan A and/or Tyrosinase melanoma markers. Furthermore, our global gene analysis also showed the upregulation of the melanocyte-differentiation gene TYRP1 and master regulator of neural crest specification SNAI2 (Slug), which is also over- expressed in aggressive melanoma. Together these findings indicate that although melanoma spheroids in our model display higher neural crest cell plasticity, they did not revert to a neural crest stem cell state and remained melanoma tumorigenic cells.

Our results indicated that melanoma spheroid cells have enhanced *in vitro* migration and invasion potential. These properties characterize aggressive tumor cells but also neural crest cells. Neural crest cells are an embryonic cell population that gives rise to cells of multiple fates and are known to migrate extremely long distance in the embryo [48]. It was strongly suggested that the high metastatic potential of melanoma cells probably emerges from the developmental origin of melanocytes in the neural crest [49]. Our global gene profiling indicated that spheroid cells from both SLM8 and Mela1 over-express genes involved in neural crest cells migration such as SNAI2 (slug), semaphorins, and ephrins. Furthermore, spheroid cells express high levels of pluripotency transcription factors. These transcription factors are expressed in neural crest cells [61], but were also found in invasive tumor cells. Expression of NANOG and Cripto-1, the co-receptor of NODAL that acts as mediator of cell fate in embryological and adult systems, was detected in aggressive melanoma [62]. NANOG [63] and OCT4 [64] were also found to be expressed in highly invasive cell populations from other tumors such as prostate cancer and glioma. It has been proposed that the migratory and invasive features of aggressive melanoma cells could be supported by cells that had reactivated elements of the molecular circuitry responsible for the specific migratory behavior of neural crest [49]. The melanoma spheroids in our model seem to behave along this theory and therefore, the occurrence of such molecular events could provide an explanation to the higher migratory and invasive capacities of these cells.

We have previously analyzed the expression of the immunological relevant molecules MHC class II on melanoma cells [22], [65], and demonstrated their involvement in melanoma protection from immune system attacks [66]. Herein, we show that melanoma spheroid cells demonstrated worse allogeneic immune stimulatory and enhanced immune-modulator functions when compared to their adherent counterparts. Given that T cells are the major fraction of PBMCs proliferating in response to allogeneic stimulation; our results suggest that melanoma spheroids in comparison with their adherent counterparts have decreased capacity to elicit proliferative response from alloreactive T cells. This possibility is in line with our data showing that melanoma spheres have enhanced capacity to inhibit proliferation of MHC-matched mitogen-activated T cells. This inhibition of T cell proliferation was accompanied with a slight increase in cell death but more importantly with a significant decrease in the percentage of cells expressing the activation marker CD25, which could indicate enhanced immune-modulator function of these spheroid cells. This could be due to the differential antigen presentation and/or to the induction of regulatory T cells. Both melanoma spheroid and adherent cells express MHC class II molecules, but melanoma spheroid cells showing more pronounced immune-modulator function express slightly lower levels of these molecules than their adherent counterparts. Melanoma cells often express various levels of MHC class II molecules, which have been associated with melanoma progression and poor prognosis [67], [68]. It has been suggested that MHC class II expression by these tumors might polarize the anti-tumor T cells response towards regulatory T cells facilitating tumor progression [69]. A recent study attributed a preferential capacity to modulate the immune response to melanoma initiating cells expressing ABCB5 and MHC class II molecules [70]. The authors showed that ABCB5^+^MHC class II^+^ cells displayed higher capacity than ABCB5- MHC class II-negative melanoma cells to inhibit IL-2-dependent T-cell activation and support the induction of CD4^+^CD25^+^FoxP3^+^ regulatory T cells. The polarization of T cell response towards regulatory T cells involves various cell-cell contact and soluble factor-mediated mechanisms. The polarization towards regulatory T cells by ABCB5^+^ melanoma initiating cells has been attributed in part to the expression of co-stimulatory CD86 by these cells [70]. However, both SLM8 and Mela1 spheroids expressed comparable levels of co-stimulatory molecules including CD86 compared to their adherent counterparts. In addition, in our model neither ABCB5+ melanoma initiating cells nor CSCs can support the enhanced immune-modulating capacity of spheroid cells, given that our spheroids were not prominently enriched with such cells. Our study thus, reveals a novel immune-modulator function of melanoma spheroid cells and suggests that in addition to melanoma initiating cells; other subpopulations could present enhanced capacity to modulate the immune response.

Our immune activation assays did not investigate the mechanism(s) through which our melanoma spheroid cells may exert their enhanced modulator effect(s) on the immune response. However, our global gene profiling underscored several candidates, which could be involved in the enhanced capacity of spheroid cells to modulate the immune response. Among these candidates, we identified IL-16 and Notch ligand Jagged 1, both over-expressed in SLM8 and Mela1 spheroid cells as potential targets. It has been shown that IL-16 produced by skin cells can inhibit alloreactive T cells proliferation inducing local immunosuppression prolonging allogeneic skin graft survival [71], and also contributes to selective regulatory T cells recruitment and expansion at inflammatory sites [47]. Notch is implicated in T cell differentiation towards the Th1, Th2, or regulatory T cells in a ligand-dependent fashion; triggering of Notch with Jagged 1 and Delta-like1, in contrast to Delta-like4 induces a dose-dependent inhibition of early activation markers CD69 and CD25 as well as inhibition of T cell proliferation [45]. The involvement of IL-16 and Jagged 1 in melanoma spheroids enhanced immune-modulator function would thus, be determined in future studies. In addition, melanoma spheroids over-express members of the semaphorin family. Growing evidence indicates that, in addition to their established role in invasion, semaphorins and their receptors are implicated in the regulation of immune response and it has been proposed that semaphorins may help cancer cells to escape immune surveillance by inhibiting T cell-mediated responses [51], [52]. Thus, semaphorins could also mediate melanoma spheroids enhanced immune-modulator function.

A recent study of glioma cells suggested that altering the differentiation state of tumor cells influence their immunosuppressive capacity [72]. The authors showed that transdifferentiation of glioma-associated cancer-initiating cells along astrocytic, neuronal, or oligodendroglial lineages decrease their immunesuppressive capacity. Although not enriched with tumor-initiating cells, melanoma spheroids also showed high transdifferentiation capacity along adipogenic and osteogenic lineages, and further studies would address whether their differentiation could impact on their immunogenic properties.

Tumor cell aggressiveness is often associated with their capacity to invade and evade immune surveillance. In this context, we showed that melanoma spheroid cells grown under neural crest conditions are endowed with important plasticity, which could indicate high capacity to respond and adapt to various microenvironmental cues, important capacity to migrate and invade, which are both essential for tumor metastasis, and immunomodulator functions suggesting enhanced capacity to evade immune surveillance. Despite their recognized relevance to tumor progression and aggressiveness, these enhanced capacities did not confer to melanoma spheroids a better capacity to form tumors in immune-compromised mice. However, an important distinction should be made between the artificial growth of human tumors in immune-compromised mice versus tumor progression in patients. The development of appropriate humanized mice models might thus, be needed to assess the involvement of these properties in melanoma progression and metastatic process as it occurs in patients. Nevertheless, melanoma spheroids constitute an appropriate model to study the aggressive molecular and functional phenotypes of melanoma cells and to identify targets that could affect their high metastatic potential and poor response to current therapies, in particular immune-based protocols.

## Supporting Information

Figure S1 **Melanoma spheroid cells display higher capacity to differentiate under mesenchymal lineages**: **A.** Adipogenic differentiation of SLM8 melanoma adherent (MA) cells and spheroid (MS) cells was assessed by Oil Red O staining (magnification ×100). **B.** Osteogenic differentiation of Mela1 adherent (MA) cells and spheroid (MS) cells was assessed by alkaline phosphatase (AP) activity detection (magnification ×10).
(TIF)

Figure S2 **Melanoma spheroids and their adherent counterparts display no side-population:** Drug efflux activity of adherent and spheroid cells was measured by their ability to exclude the Hoechst dye as previously described (Grichnik JM, 2006, J. Invest Dermatol 126:142; Goodell MA et al, 1996, J Exp Med 183:1797). Dissociated SLM8 adherent (MA) or spheroid (MS) cells were resuspended at 10^6^ cells/ml in pre-warmed DMEM medium supplemented with 3% FBS and divided into two portions. A portion was treated with 50 µM Verapamil (Verapamil (+)) and the other was left untreated (Verapamil (−)). Verapamil is a known blocker of drug efflux. Both portions were incubated in DMEM medium with 2.5 µg/ml Hoechst 33342 for 90 minutes at 37°C. After incubation the cells were washed in cold PBS and kept on ice for 5 minutes. All further proceedings were carried out at 4°C to prohibit leakage of the Hoechst dye. To discriminate dead versus live cells propidium iodide (PI) (2 µg/ml) was added to the suspended cells 5 minutes before FACS analysis for Hoechst 33342 efflux. The Hoechst 33342 dye was excited at 355 nm ultraviolet and the resultant fluorescence was measured at two wavelengths, 460 and 670 nm. Very few cells were found in the side-population (SP) that includes the cells that efficiently excluded the Hoechst dye. The number of cells (adherent and spheroid) in this region was similar when cells were incubated or not with Verapamil. Thus, SLM8 cells whether adherent or spheroid, do not display dye efflux activity. Similar results were observed with Mela1 adherent and spheroid cells.
(TIF)

Figure S3 **The expression of ABCB5 by SLM8 and Mela1 adherent and spheroid cells.** **A.** The expression of ABCB5 by SLM8 and Mela1 spheroid (MS) and adherent (MA) cells was analyzed by western blot using goat polyclonal anti-ABCB5 antibody (ABCB5 (N-13): sc-104019, Santa Cruz Biotechnology) detecting a fragment of approximately 89 kDa. Extracts from Skmel5 melanoma cells and K562 leukemia cells both reported to be positive for ABCB5 by real-time PCR (Frank et al, Cancer Res, 65:4320, 2005; Lehne et al, Leuk Res, 33:1379, 2009) were used as controls. Re-blotting with anti-b-actin ensured equal loading. Similar results were obtained when western blot analysis was carried out using goat polyclonal anti-ABCB5 from ProSci (46–620) (Yang et al, 2010, BMC Cancer, 10:338). **B.** The expression of ABCB5 by adherent and spheroid cells as analyzed by immunostaining. Dissociated cells from SLM8-MA and SLM8-MS were incubated with goat polyclonal anti-ABCB5 antibody (ABCB5 (N-13): sc-104019, Santa Cruz Biotechnology) or with IgG isotype control, then with FITC-conjugated donkey anti-goat IgG (Santa Cruz Biotechnologies). Cells were then cytocentrifuged onto slides (cytospin) and mounted in VECTASHIELD Mounting Media with DAPI. Images were acquired by immunofluorescence microscopy on a Zeiss Axiovert 200 M microscope (Zeiss, Germany) equipped with a Plan Apochromat 63× N.A.1.4 oil-immersion objective and a Axiocam MRM camera (Zeiss) using the Axiovision v4.5.0.0 software (Zeiss). Similar results were obtained with Mela1 cells. **C.** The expression of ABCB5 in adherent and spheroid cells as analyzed by flow cytometry. Dissociated cells from SLM8-MA and SLM8-MS were incubated with 10 mg/ml rabbit polyclonal anti-ABCB5 antibody (clone RB16781, Abgent Inc, San Diago, CA) or IgG isotype control, then with PE-conjugated goat anti-rabbit IgG (Abgent). Expression was determined by Canto II flow cytometer (BD Biosciences) and analyzed by Diva and FlowJo softwares. WM852 melanoma cells were used as positive control (Abgent, data courtesy of Dr Steve Reuland, University of Colorado, Denver). Red unfilled histograms (ABCB5) versus black unfilled histograms for respective isotype control.
(TIF)

Figure S4 **Melanoma spheroid and adherent cells have similar tumorigenic capacity.** The capacity of SLM8 and Mela1 adherent and spheroid cells to form tumors was determined by subcutaneously injecting groups of 5-week-old female nude mice (n = 3/group) with single-cell inoculates of adherent or spheroid cells. A log-fold range from cell doses unable to efficiently initiate tumors (10^4^ cells) to doses reported to consistently initiate tumors (10^6^ cells) when melanoma spheroid or melanoma-initiating cells (Fang, et al., Cancer Res, 2005, 65:9328; Monzani, et al., Eur J Cancer, 2007, 43:935)(Schatton, et al., Nature, 2008, 451:345) were transplanted into immune-compromised mice models were assayed. Tumor formation/growth was monitored for the duration of the experiments or until disease state required euthanasia. For secondary tumor assays, adherent or spheroid cells-induced primary tumors were removed after euthanasia at day 90, minced and subjected to enzymatic digestion. Adherent or spheroid cell-tumor derived single cells were then injected and monitored as above. Tumor diameters were measured with calipers and tumor volume was calculated as (Width)^2^×(Length)×0.5 (Hoek, et al., Cancer Res, 2008, 68:650). **A.** Incidence of primary and secondary tumors. Injection of melanoma adherent or spheroid cells from SLM8 or Mela1 at 10^6^ led to the growth of primary tumors in all mice. Primary tumors were also formed with 1/3 mice injected with 10^5^ adherent or spheroid cells from Mela1 but not SLM8, while 10^4^ cells inoculates failed to initiate any primary tumors. Injection of log-fold inoculates of single-cell suspensions from adherent or spheroid cells-induced primary tumors resulted in secondary tumors formation at 10^6^ and 10^5^. **B.** Growth curves of SLM8 adherent or spheroid cells-induced primary and secondary tumors. Tumor growth curves were established by monitoring the volume of both primary and secondary tumors and are presented as mean values of tumors volumes obtained with each mice (n = 3) ± SD. Adherent and spheroid cells-induced primary tumors appeared as small nodules and reached 100 mm^3^ between 30–40 days (upper panel). For both adherent and spheroid cells secondary tumors appeared earlier compared with the primary ones and reached 100 mm^3^ within only 20 days of injection (lower panels). Similar data were obtained with tumors induced after injection with Mela1 adherent and spheroid cells (data not shown). Thus, in our melanoma model, spheroid and adherent cells behave similarly and show similar tumor formation capacity.
(TIF)

Figure S5 **Common regulated genes in spheroid versus adherent cells between Mela1 and SLM8 cells.**
(TIF)

Table S1 **A.** Commonly up-regulated genes in Mela1 and SLM8. **B.** Commonly down- regulated genes in Mela1 and SLM8. **C.** Oppositely-regulated genes in Mela1 and SLM8.
(PDF)

Table S2 **Developmental processes regulated genes.** List of the genes regulated in melanoma spheroids (MS)/adherent cells (MA) involved in developmental processes along with their respective fold-changes as determined by biological processes using PANTHER software.
(PDF)

Table S3 **Ectoderm, neurogenesis, mesoderm and skeletal regulated genes.** List of the genes regulated in melanoma spheroids (MS)/adherent cells (MA) involved in ectoderm, development, neurogenesis, mesoderm and skeletal development processes along with their respective fold-changes as determined by biological processes using PANTHER software.
(PDF)

Table S4 **Adhesion/communication regulated genes.** List of the genes regulated in melanoma spheroids (MS)/adherent cells (MA) involved in cell adhesion/communication processes along with their respective fold-changes as determined by biological processes using PANTHER software.
(PDF)

Table S5 **Structure/motility regulated genes.** List of the genes regulated in melanoma spheroids (MS)/adherent cells (MA) involved in cell structure/motility processes along with their respective fold-changes as determined by biological processes using PANTHER software.
(PDF)

Table S6 **Immunity and defense.** List of the genes regulated in melanoma spheroids (MS)/adherent cells (MA) involved in immunity and defence processes along with their respective fold-changes as determined by biological processes using PANTHER software.
(PDF)

Table S7 **Molecular functions regulated genes.** List of the ECM, cell adhesion, cytoskeleton proteins, actin-binding and non-motor actin-binding regulated genes in melanoma spheroids (MS)/adherent cells (MA) along with their respective fold-changes as determined by molecular functions analysis using PANTHER software.
(PDF)

Table S8 **Molecular functions regulated genes.** List of the receptors, other receptors and membrane-bound signaling genes in melanoma spheroids (MS)/adherent cells (MA) along with their respective fold-changes as determined by molecular functions analysis using PANTHER software.
(PDF)

Table S9 **Signaling pathways regulated genes.** List of the genes regulated in melanoma spheroids (MS)/adherent cells (MA) involved in Notch, endothelin and axon guidance signaling along with their respective fold-changes as determined by signaling pathways analysis using PANTHER software.
(PDF)

Table S10 **Nucleotide sequence of quantitative PCR primers and corresponding annealing temperatures.**
(PDF)

Table S11 **Nucleotide sequence of semi-quantitative PCR primers, corresponding annealing temperatures and number of cycles performed.**
(PDF)
