# Supplementary figures and images for: Melanoma Spheroids Grown Under Neural Crest Cell Conditions Are Highly Plastic Migratory/Invasive Tumor Cells Endowed with Immunomodulator Function
Source: PLoS One. 2011 Apr 15;6(4):e18784. doi: 10.1371/journal.pone.0018784 (PMC3078142; doi:10.1371/journal.pone.0018784)

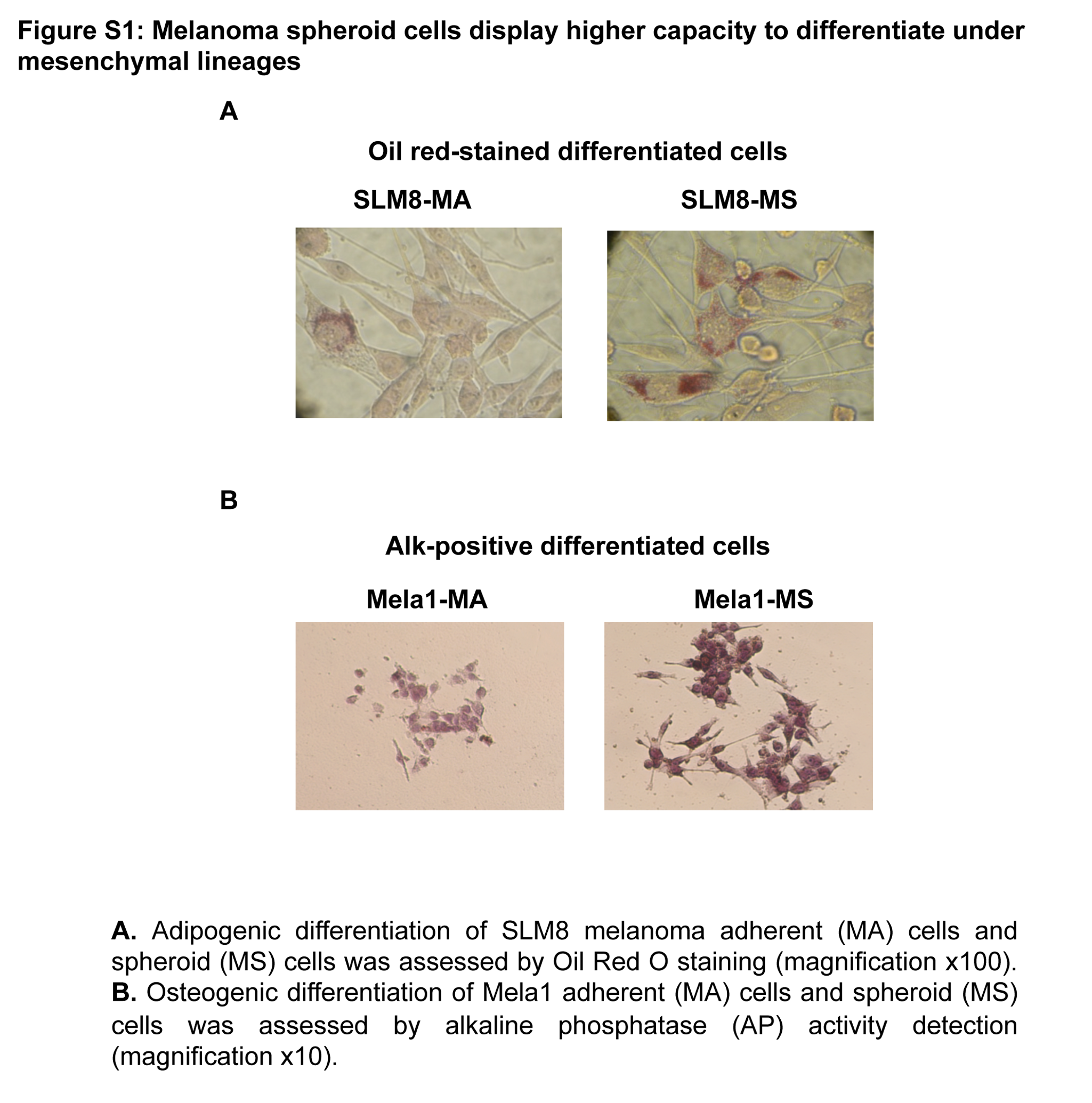

Supplement: Figure S1 — Melanoma spheroid cells display higher capacity to differentiate under mesenchymal lineages: A. Adipogenic differentiation of SLM8 melanoma adherent (MA) cells and spheroid (MS) cells was assessed by Oil Red O staining (magnification ×100). B. Osteogenic differentiation of Mela1 adherent (MA) cells and spheroid (MS) cells was assessed by alkaline phosphatase (AP) activity detection (magnification ×10). (TIF) [file pone.0018784.s001.tif]

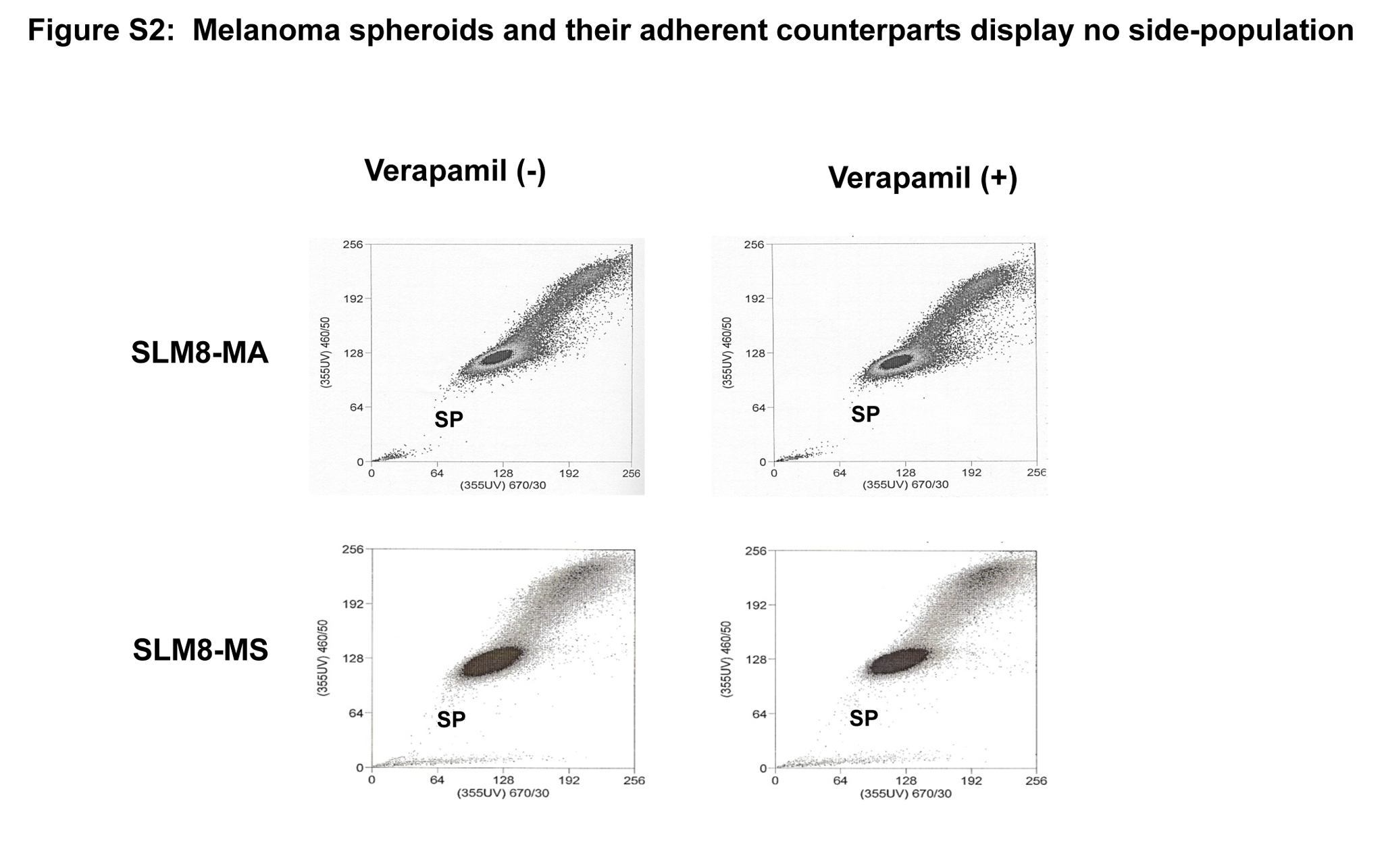

Supplement: Figure S2 — Melanoma spheroids and their adherent counterparts display no side-population: Drug efflux activity of adherent and spheroid cells was measured by their ability to exclude the Hoechst dye as previously described (Grichnik JM, 2006, J. Invest Dermatol 126:142; Goodell MA et al, 1996, J Exp Med 183:1797). Dissociated SLM8 adherent (MA) or spheroid (MS) cells were resuspended at 106 cells/ml in pre-warmed DMEM medium supplemented with 3% FBS and divided into two portions. A portion was treated with 50 µM Verapamil (Verapamil (+)) and the other was left untreated (Verapamil (−)). Verapamil is a known blocker of drug efflux. Both portions were incubated in DMEM medium with 2.5 µg/ml Hoechst 33342 for 90 minutes at 37°C. After incubation the cells were washed in cold PBS and kept on ice for 5 minutes. All further proceedings were carried out at 4°C to prohibit leakage of the Hoechst dye. To discriminate dead versus live cells propidium iodide (PI) (2 µg/ml) was added to the suspended cells 5 minutes before FACS analysis for Hoechst 33342 efflux. The Hoechst 33342 dye was excited at 355 nm ultraviolet and the resultant fluorescence was measured at two wavelengths, 460 and 670 nm. Very few cells were found in the side-population (SP) that includes the cells that efficiently excluded the Hoechst dye. The number of cells (adherent and spheroid) in this region was similar when cells were incubated or not with Verapamil. Thus, SLM8 cells whether adherent or spheroid, do not display dye efflux activity. Similar results were observed with Mela1 adherent and spheroid cells. (TIF) [file pone.0018784.s002.tif]

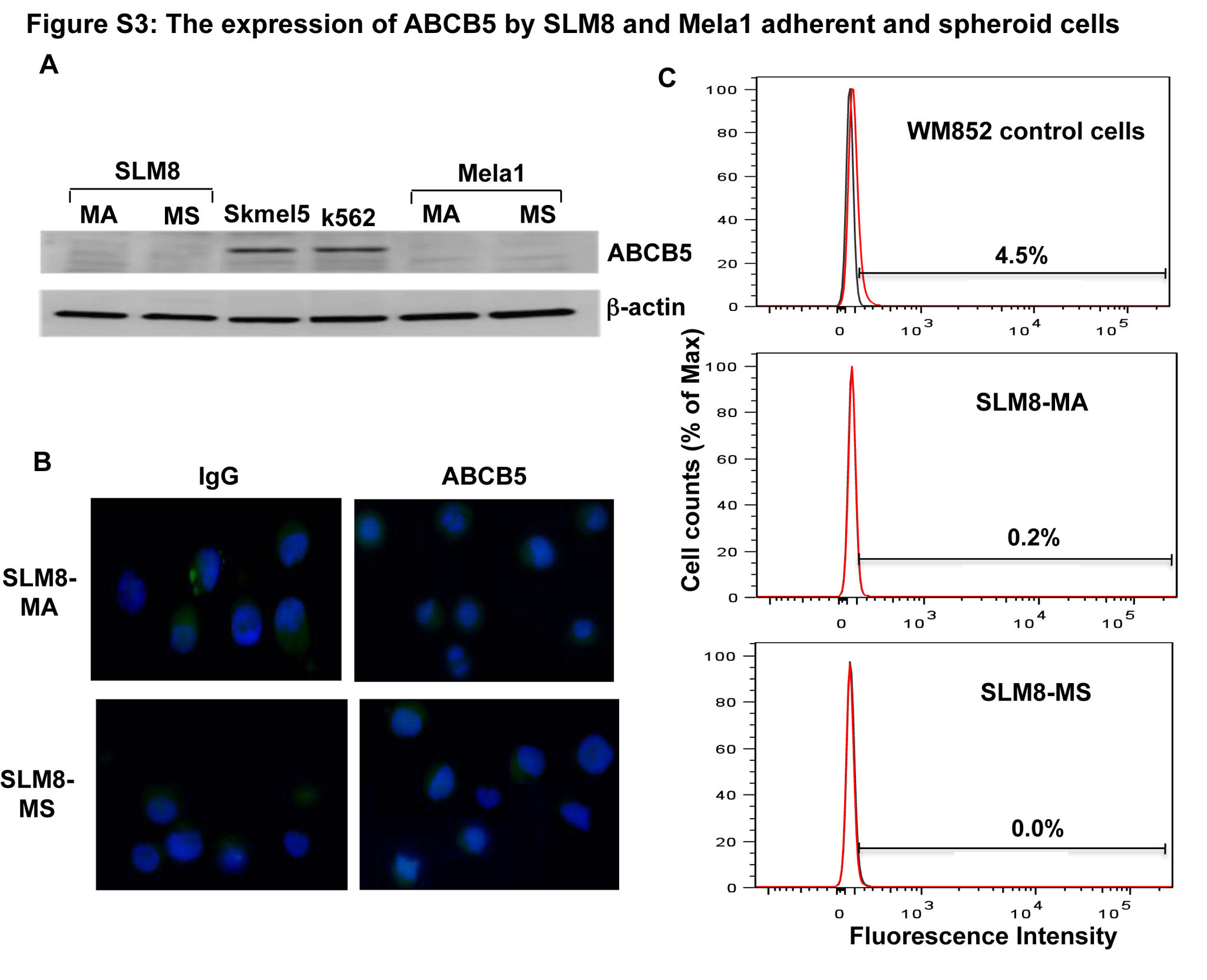

Supplement: Figure S3 — The expression of ABCB5 by SLM8 and Mela1 adherent and spheroid cells. A. The expression of ABCB5 by SLM8 and Mela1 spheroid (MS) and adherent (MA) cells was analyzed by western blot using goat polyclonal anti-ABCB5 antibody (ABCB5 (N-13): sc-104019, Santa Cruz Biotechnology) detecting a fragment of approximately 89 kDa. Extracts from Skmel5 melanoma cells and K562 leukemia cells both reported to be positive for ABCB5 by real-time PCR (Frank et al, Cancer Res, 65:4320, 2005; Lehne et al, Leuk Res, 33:1379, 2009) were used as controls. Re-blotting with anti-b-actin ensured equal loading. Similar results were obtained when western blot analysis was carried out using goat polyclonal anti-ABCB5 from ProSci (46–620) (Yang et al, 2010, BMC Cancer, 10:338). B. The expression of ABCB5 by adherent and spheroid cells as analyzed by immunostaining. Dissociated cells from SLM8-MA and SLM8-MS were incubated with goat polyclonal anti-ABCB5 antibody (ABCB5 (N-13): sc-104019, Santa Cruz Biotechnology) or with IgG isotype control, then with FITC-conjugated donkey anti-goat IgG (Santa Cruz Biotechnologies). Cells were then cytocentrifuged onto slides (cytospin) and mounted in VECTASHIELD Mounting Media with DAPI. Images were acquired by immunofluorescence microscopy on a Zeiss Axiovert 200 M microscope (Zeiss, Germany) equipped with a Plan Apochromat 63× N.A.1.4 oil-immersion objective and a Axiocam MRM camera (Zeiss) using the Axiovision v4.5.0.0 software (Zeiss). Similar results were obtained with Mela1 cells. C. The expression of ABCB5 in adherent and spheroid cells as analyzed by flow cytometry. Dissociated cells from SLM8-MA and SLM8-MS were incubated with 10 mg/ml rabbit polyclonal anti-ABCB5 antibody (clone RB16781, Abgent Inc, San Diago, CA) or IgG isotype control, then with PE-conjugated goat anti-rabbit IgG (Abgent). Expression was determined by Canto II flow cytometer (BD Biosciences) and analyzed by Diva and FlowJo softwares. WM852 melanoma cells were used as positive con [file pone.0018784.s003.tif]

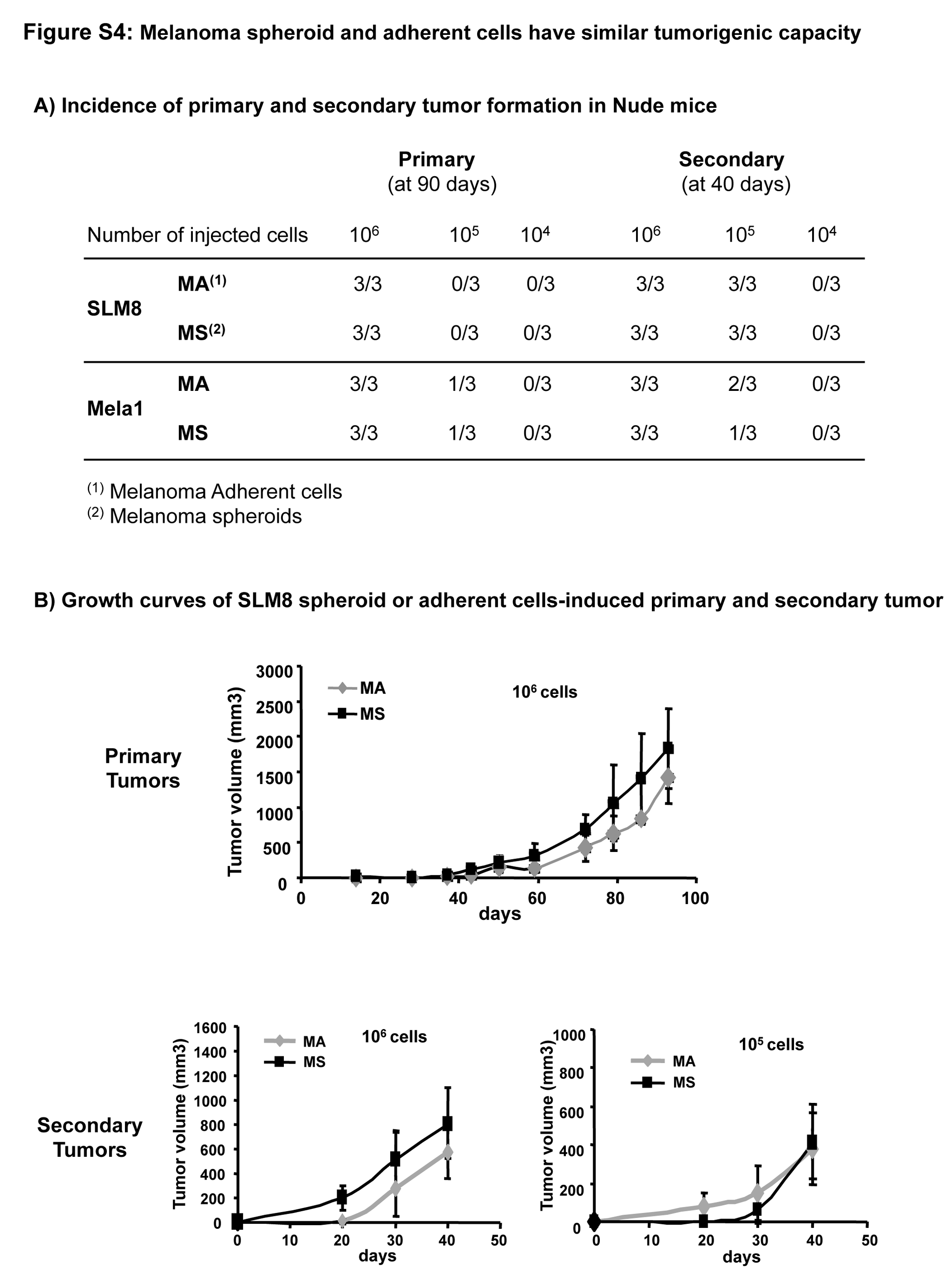

Supplement: Figure S4 — Melanoma spheroid and adherent cells have similar tumorigenic capacity. The capacity of SLM8 and Mela1 adherent and spheroid cells to form tumors was determined by subcutaneously injecting groups of 5-week-old female nude mice (n = 3/group) with single-cell inoculates of adherent or spheroid cells. A log-fold range from cell doses unable to efficiently initiate tumors (104 cells) to doses reported to consistently initiate tumors (106 cells) when melanoma spheroid or melanoma-initiating cells (Fang, et al., Cancer Res, 2005, 65:9328; Monzani, et al., Eur J Cancer, 2007, 43:935)(Schatton, et al., Nature, 2008, 451:345) were transplanted into immune-compromised mice models were assayed. Tumor formation/growth was monitored for the duration of the experiments or until disease state required euthanasia. For secondary tumor assays, adherent or spheroid cells-induced primary tumors were removed after euthanasia at day 90, minced and subjected to enzymatic digestion. Adherent or spheroid cell-tumor derived single cells were then injected and monitored as above. Tumor diameters were measured with calipers and tumor volume was calculated as (Width)2×(Length)×0.5 (Hoek, et al., Cancer Res, 2008, 68:650). A. Incidence of primary and secondary tumors. Injection of melanoma adherent or spheroid cells from SLM8 or Mela1 at 106 led to the growth of primary tumors in all mice. Primary tumors were also formed with 1/3 mice injected with 105 adherent or spheroid cells from Mela1 but not SLM8, while 104 cells inoculates failed to initiate any primary tumors. Injection of log-fold inoculates of single-cell suspensions from adherent or spheroid cells-induced primary tumors resulted in secondary tumors formation at 106 and 105. B. Growth curves of SLM8 adherent or spheroid cells-induced primary and secondary tumors. Tumor growth curves were established by monitoring the volume of both primary and secondary tumors and are presented as mean values of tumors volumes obtained with each mice ( [file pone.0018784.s004.tif]

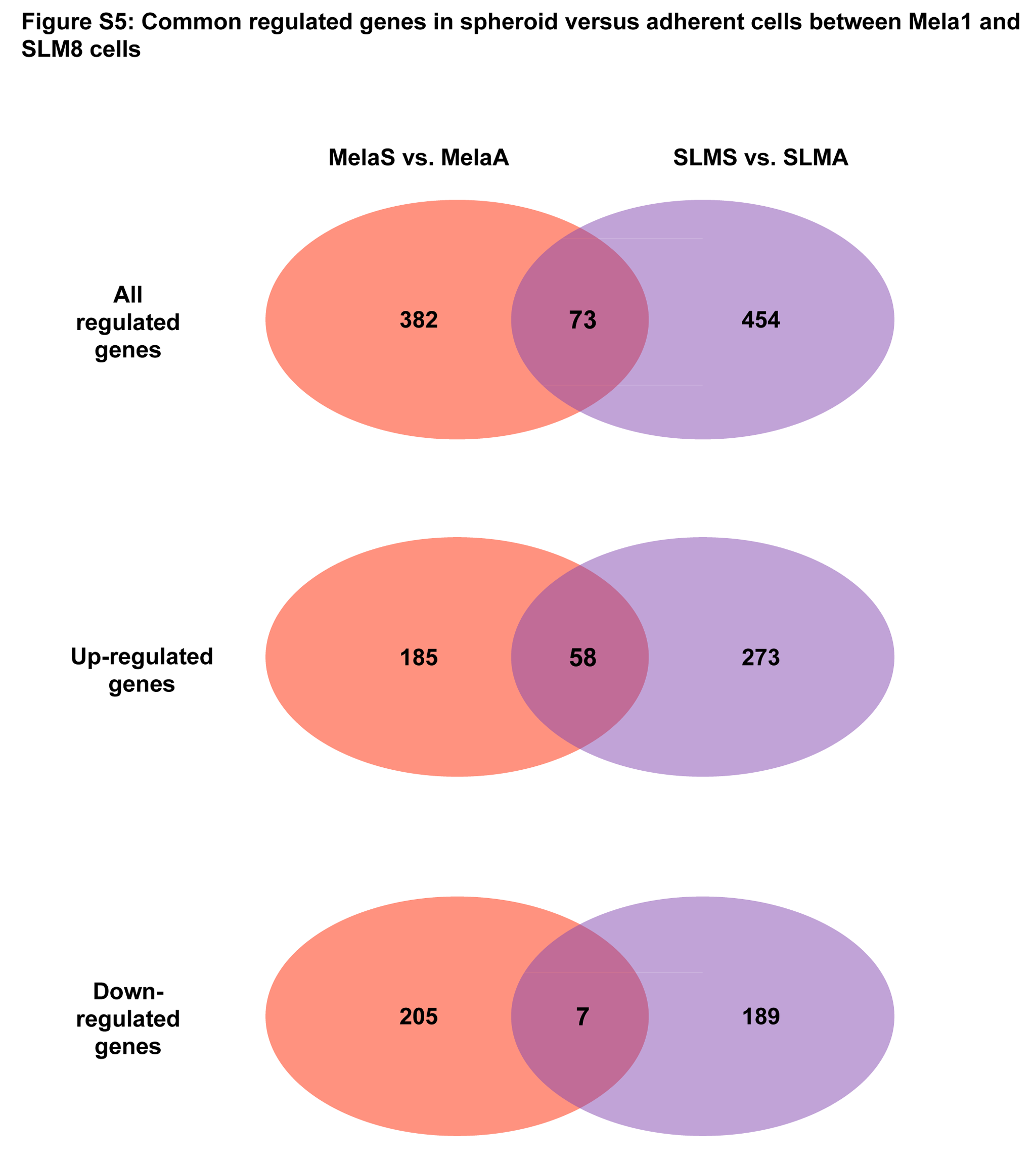

Supplement: Figure S5 — Common regulated genes in spheroid versus adherent cells between Mela1 and SLM8 cells. (TIF) [file pone.0018784.s005.tif]
