# Supplementary material for: Melanoma Spheroids Grown Under Neural Crest Cell Conditions Are Highly Plastic Migratory/Invasive Tumor Cells Endowed with Immunomodulator Function
Source: PLoS One. 2011 Apr 15;6(4):e18784. doi: 10.1371/journal.pone.0018784 (PMC3078142; doi:10.1371/journal.pone.0018784)
Supplement: Table S1 — A. Commonly up-regulated genes in Mela1 and SLM8. B. Commonly down- regulated genes in Mela1 and SLM8. C. Oppositely-regulated genes in Mela1 and SLM8. (PDF) [file pone.0018784.s006.pdf]

Table S1 A: Commonly up-regulated genes in Mela1 and SLM8

FAST DB ID	Gene Symbol	Gene Name	Mela1-MS vs Mela1-MA	SLM8-MS vs SLM-MA
9985	TGFBI	transforming growth factor, beta-induced, 68kDa	up	up
1351	ADAMTS1	ADAM metallopeptidase with thrombospondin type 1 motif, 1	up	up
17860	CNIH3	cornichon homolog 3 (Drosophila)	up	up
11209	LSAMP	limbic system-associated membrane protein	up	up
9033	ACSS2	acyl-CoA synthetase short-chain family member 2	up	up
2307	TYRP1	tyrosinase-related protein 1	up	up
7203	DTX4	deltex 4 homolog (Drosophila)	up	up
16665	TNFAIP3	tumor necrosis factor, alpha-induced protein 3	up	up
6931	VCAN	versican	up	up
15792	FASN	fatty acid synthase	up	up
2559	SLC6A6	solute carrier family 6 (neurotransmitter transporter, taurine), member 6	up	up
2563	SCUBE2	signal peptide, CUB domain, EGF-like 2	up	up
8848	UNC13A	unc-13 homolog A (C. elegans)	up	up
11068	CTTNBP2	cortactin binding protein 2	up	up
8533	SQLE	squalene epoxidase	up	up
12627	RUNX2	runt-related transcription factor 2	up	up
16134	RAB17	RAB17, member RAS oncogene family	up	up
13313	RNF43	ring finger protein 43	up	up
11004	PCSK6	proprotein convertase subtilisin/kexin type 6	up	up
8212	DEPDC6	DEP domain containing 6	up	up
9607	SLC7A11	solute carrier family 7, (cationic amino acid transporter, y+ system) member 11	up	up
10744	LAMB1	laminin, beta 1	up	up
12104	HOXB13	homeobox B13	up	up
13058	---	---	up	up
8757	MGAT4A	mannosyl (alpha-1,3-)-glycoprotein beta-1,4-N-acetylglucosaminyltransferase, isozyme A	up	up
12422	PLTP	phospholipid transfer protein	up	up
8365	GDF11	growth differentiation factor 11	up	up
3366	CHN2	chimerin (chimaerin) 2	up	up
1289	ODC1	ornithine decarboxylase 1	up	up
4669	CHRNA6	cholinergic receptor, nicotinic, alpha 6	up	up
16655	LPHN2	latrophilin 2	up	up
16542	C6orf192	chromosome 6 open reading frame 192	up	up
18059	ARHGEF9	Cdc42 guanine nucleotide exchange factor (GEF) 9	up	up
12110	TTLL6	tubulin tyrosine ligase-like family, member 6	up	up
7933	FADS2	fatty acid desaturase 2	up	up
12434	MIA	melanoma inhibitory activity	up	up
12722	ATP10B	ATPase, class V, type 10B	up	up
13882	NCAM1	neural cell adhesion molecule 1	up	up
11178	SLC6A15	solute carrier family 6, member 15	up	up
6056	TRAF3	TNF receptor-associated factor 3	up	up
14626	SORL1	sortilin-related receptor, L(DLR class) A repeats-containing	up	up
6759	ACP5	acid phosphatase 5, tartrate resistant	up	up
14226	P2RX7	purinergic receptor P2X, ligand-gated ion channel, 7	up	up
12009	DLL3	delta-like 3 (Drosophila)	up	up
17651	IVNS1ABP	influenza virus NS1A binding protein	up	up
8276	HAS2	hyaluronan synthase 2	up	up
7073	ACSS1	acyl-CoA synthetase short-chain family member 1	up	up
883	TNFRSF19	tumor necrosis factor receptor superfamily, member 19	up	up
3317	CREB5	cAMP responsive element binding protein 5	up	up
16692	SORCS1	sortilin-related VPS10 domain containing receptor 1	up	up
11556	PLXNC1	plexin C1	up	up
14068	SIDT2	SID1 transmembrane family, member 2	up	up
9218	IL16	interleukin 16 (lymphocyte chemoattractant factor)	up	up
7398	TIMP1	TIMP metallopeptidase inhibitor 1	up	up
5669	JAG1	jagged 1 (Alagille syndrome)	up	up
5043	SNAI2	snail homolog 2 (Drosophila)	up	up
4887	LCP1	lymphocyte cytosolic protein 1 (L-plastin)	up	up
12030	SDC4	syndecan 4	up	up

Table S1 B: Commonly down-regulated genes in Mela1 and SLM8

FAST DB ID	Gene Symbol	Gene Name	Mela1-MS vs Mela1-MA	SLM8-MS vs SLM8-MA
2429	ABCB5	ATP-binding cassette, sub-family B (MDR/TAP), member 5	down	down
15344	IFIT3	interferon-induced protein with tetratricopeptide repeats 3	down	down
10288	KIF20A	kinesin family member 20A	down	down
11298	MGAT5	mannosyl (alpha-1,6-)-glycoprotein beta-1,6-N-acetyl-glucosaminyltransferase	down	down
6323	IMPA1	inositol(myo)-1(or 4)-monophosphatase 1	down	down
9066	GDF15	growth differentiation factor 15	down	down
211	PYGL	phosphorylase, glycogen; liver (Hers disease, glycogen storage disease type VI)	down	down

Table S1 C: Oppositely regulated genes in Mela1 and SLM8

FAST DB ID	Gene Symbol	Gene Name	Mela1-MS vs Mela1-MA	SLM8-MS vs SLM8-MA
8795	RORB	RAR-related orphan receptor B	up	down
8995	FLNB	filamin B, beta (actin binding protein 278)	down	up
14953	SLC2A1	solute carrier family 2 (facilitated glucose transporter), member 1	down	up
1459	ENO2	enolase 2 (gamma, neuronal)	down	up
15717	FER1L3	fer-1-like 3, myoferlin (C. elegans)	down	up
5912	PLOD1	procollagen-lysine 1, 2-oxoglutarate 5-dioxygenase 1	down	up
17105	SLC16A1	solute carrier family 16, member 1 (monocarboxylic acid transporter 1)	down	up
9005	ECE1	endothelin converting enzyme 1	down	up
