# Supplementary material for: Melanoma Spheroids Grown Under Neural Crest Cell Conditions Are Highly Plastic Migratory/Invasive Tumor Cells Endowed with Immunomodulator Function
Source: PLoS One. 2011 Apr 15;6(4):e18784. doi: 10.1371/journal.pone.0018784 (PMC3078142; doi:10.1371/journal.pone.0018784)
Supplement: Table S2 — Developmental processes regulated genes. List of the genes regulated in melanoma spheroids (MS)/adherent cells (MA) involved in developmental processes along with their respective fold-changes as determined by biological processes using PANTHER software. (PDF) [file pone.0018784.s007.pdf]

Table S2: Developmental processes regulated genes

Up Mela1 MS/MA fold	Developmental processes
15.28	TGFBI
6.83	DNER
5.40	PLXNC1
5.05	JAG1
4.94	EPHA3
4.75	DLL3
4.58	NCAM1
4.37	RORB
4.25	SCML1
3.73	PCSK6
3.42	BMP7
3.30	TNFRSF19
3.24	TIMP1
3.21	ERBB4
2.97	LSAMP
2.82	CNIH3
2.75	MET
2.66	EYA4
2.57	THBS2
2.52	SMAD9
2.38	PYGO1
2.30	RUNX2
2.27	DTX4
2.25	PTPRZ1
2.25	HMCN1
2.23	SDC4
2.19	ROBO2
2.16	GDF11
2.13	SCUBE2
2.12	ADAMTS1
2.09	KIT
2.07	SEMA5A
2.02	CRB1
1.94	THBS1
1.78	ACP5
1.72	PELO
1.62	EPHA4
1.57	PRKACB
1.55	IL16
1.48	GAB1
1.48	ADAM23
1.43	MERTK
1.39	POR
No. genes >1.5	39
1.5> no. >1.2	4
Total	43

Down Mela1 MS/MA fold	Developmental processes
4.34	ID3
3.83	MCAM
2.59	GDF15
2.50	SEMA3A
2.45	LMO4
2.34	TES
2.15	TTF2
2.03	SKIL
2.03	KIF2C
1.88	EPAS1
1.87	KIF20A
1.85	EYA3
1.77	CAMK4
1.65	HS2ST1
1.62	NOTCH2
1.57	ZFR
1.57	MFN2
1.55	TRAK2
1.51	PARD6B
1.51	MSH2
1.51	RARB
1.48	ABLIM3
1.34	LRIG2
1.30	WDR35
1.29	IFRD1
No. genes >1.5	21
1.5> no. >1.2	4
Total	25

Up SLM8 MS/MA fold	Developmental processes
7.14	MGP
4.82	TGFBI
3.85	ADAMTS1
3.82	CNIH3
3.64	L1CAM
3.35	CUBN
3.35	LSAMP
3.29	DUSP1
3.15	PDK1
3.07	DTX4
2.99	PLK2
2.53	SEMA3D
2.52	TNS1
2.45	SCUBE2
2.44	KLF4
2.25	RUNX2
2.21	EDIL3
2.19	PCSK6
2.08	RORA
2.05	KDR
1.99	EPHA2
1.95	GDF11
1.89	PBX4
1.86	TNC
1.77	PAN2
1.77	FABP3
1.76	NRCAM
1.76	LDLR
1.75	DLG4
1.70	VLDLR
1.65	NCAM1
1.64	LRP1
1.64	HOXB9
1.63	FGFR1
1.60	ACP5
1.59	DLL3
1.56	MORF4L2
1.54	TNFRSF19
1.53	LDLRAD3
1.48	LRP6
1.44	PAX9
1.44	LAMA1
1.42	RSF1
1.42	CDON
1.41	DZIP1
1.39	DIP2C
1.39	NEO1
1.37	PLXNC1
1.36	NFKB2
1.36	IL16
1.35	SMAD3
1.35	TIMP1
1.35	JAG1
1.34	GRN
1.33	MLL
1.28	DCT
1.27	CRIP2
1.20	SDC4
No. genes >1.5	39
1.5> no. >1.2	19
Total	58

Dowm SLM8 MS/MA fold	Developmental processes
2.45	KIF20A
2.34	LRP2
2.17	PLK1
2.06	BUB1B
1.89	PHF19
1.88	DLC1
1.76	MAMDC2
1.67	HEY2
1.54	PPP1CA
1.51	ABL1
1.40	SBNO2
1.36	GDF15
1.34	RB1
1.31	ABLIM1
1.29	RORB
1.28	CHM
No. genes >1.5	10
1.5> no. >1.2	6
Total	16
