# Supplementary material for: Melanoma Spheroids Grown Under Neural Crest Cell Conditions Are Highly Plastic Migratory/Invasive Tumor Cells Endowed with Immunomodulator Function
Source: PLoS One. 2011 Apr 15;6(4):e18784. doi: 10.1371/journal.pone.0018784 (PMC3078142; doi:10.1371/journal.pone.0018784)
Supplement: Table S3 — Ectoderm, neurogenesis, mesoderm and skeletal regulated genes. List of the genes regulated in melanoma spheroids (MS)/adherent cells (MA) involved in ectoderm, development, neurogenesis, mesoderm and skeletal development processes along with their respective fold-changes as determined by biological processes using PANTHER software. (PDF) [file pone.0018784.s008.pdf]

Table S3: Ectoderm, Neurogenesis, Mesoderm, and skeletal regulated genes

Up Mela1 MS/MA fold	Ectoderm	Neuro-genesis
6.83	DNER	DNER
5.05	JAG1	JAG1
4.94	EPHA3	EPHA3
4.75	DLL3	DLL3
4.58	NCAM1	NCAM1
2.97	LSAMP	LSAMP
2.38	PYGO1	PYGO1
2.27	DTX4	DTX4
2.19	ROBO2	ROBO2
2.12	ADAMTS1	ADAMTS1
2.07	SEMA5A	SEMA5A
2.02	CRB1	CRB1
1.62	EPHA4	EPHA4
1.55	IL16	IL16
No. genes >1.5	14	14
1.5> no. >1.2	0	0
Total	14	14

Up SLM8 MS/MA fold	Ectoderm	Neuro-genesis
3.85	ADAMTS1	ADAMTS1
3.64	L1CAM	L1CAM
3.35	CUBN	CUBN
3.35	LSAMP	LSAMP
3.07	DTX4	DTX4
2.53	SEMA3D	SEMA3D
1.99	EPHA2	EPHA2
1.86	TNC	TNC
1.77	PAN2	PAN2
1.77	FABP3	
1.76	NRCAM	NRCAM
1.75	DLG4	DLG4
1.65	NCAM1	NCAM1
1.64	HOXB9	HOXB9
1.63	FGFR1	FGFR1
1.59	DLL3	DLL3
1.39	DIP2C	DIP2C
1.39	NEO1	NEO1
1.36	IL16	IL16
1.35	JAG1	JAG1
1.28	DCT	
No. genes >1.5	16	16
1.5> no. >1.2	5	4
Total	21	20

Up Mela1 MS/MA fold	Mesoderm	Skeletal
15.28	TGFBI	TGFBI
4.94	EPHA3	
3.73	PCSK6	
3.42	BMP7	BMP7
2.30	RUNX2	RUNX2
2.23	SDC4	SDC4
2.16	GDF11	
2.13	SCUBE2	SCUBE2
2.12	ADAMTS1	ADAMTS1
1.78	ACP5	ACP5
1.62	EPHA4	
1.48	GAB1	
No. genes >1.5	11	7
1.5> no. >1.2	1	
Total	12	7

Up SLM8 MS/MA fold	Mesoderm	Skeletal
7.14	MGP	MGP
4.82	TGFBI	TGFBI
3.85	ADAMTS1	ADAMTS1
3.29	DUSP1	
2.53	SEMA3D	
2.52	TNS1	
2.45	SCUBE2	SCUBE2
2.44	KLF4	
2.25	RUNX2	RUNX2
2.21	EDIL3	
2.19	PCSK6	
2.05	KDR	
1.99	EPHA2	
1.95	GDF11	
1.89	PBX4	
1.86	TNC	
1.60	ACP5	ACP5
1.44	PAX9	PAX9
1.42	CDON	
1.27	CRIP2	
1.20	SDC4	SDC4
No. genes >1.5	17	6
1.5> no. >1.2	4	2
Total	21	8

Down Mela1 MS/MA fold	Ectoderm	Neuro-genesis
2.50	SEMA3A	SEMA3A
2.45	LMO4	LMO4
2.15	TTF2	TTF2
1.88	EPAS1	EPAS1
1.77	CAMK4	CAMK4
1.62	NOTCH2	NOTCH2
1.55	TRAK2	TRAK2
1.48	ABLIM3	ABLIM3
1.30	WDR35	WDR35
1.29	IFRD1	IFRD1
No. genes >1.5	7	7
1.5> no. >1.2	3	3
Total	10	10

Dowm SLM8 MS/MA fold	Ectoderm	Neuro-genesis
1.67	HEY2	HEY2
1.31	ABLIM1	ABLIM1
No. genes >1.5	1	1
1.5> no. >1.2	1	1
Total	2	2

Down Mela1 MS/MA fold	Mesoderm	Skeletal
2.59	GDF15	GDF15
2.50	SEMA3A	
2.45	LMO4	
2.15	TTF2	
2.03	SKIL	
No. genes >1.5	5	1
1.5> no. >1.2	0	0
Total	5	1

Dowm SLM8 MS/MA fold	Mesoderm	Skeletal
1.36	GDF15	GDF15
No. genes >1.5	0	0
1.5> no. >1.2	1	1
Total	1	1
