# Supplementary material for: Melanoma Spheroids Grown Under Neural Crest Cell Conditions Are Highly Plastic Migratory/Invasive Tumor Cells Endowed with Immunomodulator Function
Source: PLoS One. 2011 Apr 15;6(4):e18784. doi: 10.1371/journal.pone.0018784 (PMC3078142; doi:10.1371/journal.pone.0018784)
Supplement: Table S4 — Adhesion/communication regulated genes. List of the genes regulated in melanoma spheroids (MS)/adherent cells (MA) involved in cell adhesion/communication processes along with their respective fold-changes as determined by biological processes using PANTHER software. (PDF) [file pone.0018784.s009.pdf]

Table S4: Adhesion/Communication regulated genes

Up Mela1 MS/MA fold	Cell adhesion	Cell commu-nication	Cell adhesion signaling	ECM signaling
41.89		CCL18		
15.28	TGFBI	TGFBI		TGFBI
6.83		DNER		
4.75		DLL3		
4.58	NCAM1	NCAM1	NCAM1	
4.31		CHRNA6		
3.35	LAMA4	LAMA4	LAMA4	LAMA4
3.30		TNFRSF19		
2.97	LSAMP			
2.75		MET		
2.68		MAGI1		
2.52		SMAD9		
2.45	GRIP1	GRIP1		
2.40		TMTC1		
2.38		IL6R		
2.37		ADAMTS3	ADAMTS3	
2.30	PCOLCE			
2.25	PTPRZ1	PTPRZ1		
2.25	HMCN1			
2.23	SDC4			
2.19	ROBO2			
2.14	ITGA7			
2.13		SCUBE2	SCUBE2	
2.12		ADAMTS1	ADAMTS1	ADAMTS1
2.07		SEMA5A		
2.02		CRB1		
1.85		LAMC1	LAMC1	LAMC1
1.71	CD96	CD96		
1.57		PRKACB		
1.51	ITGA9			
1.50		LAMB1	LAMB1	LAMB1
1.48		ADAM23	ADAM23	
1.47		CORO1C		
1.39	POR			
No. genes >1.5	13	25	7	5
1.5> no. >1.2	1	2	1	0
Total	14	27	8	5

Down Mela1 MS/MA fold	Cell adhesion	Cell commu-nication	Cell adhesion signaling	ECM signaling
3.83	MCAM			
2.91		MME		
2.59		GDF15		
2.50		SEMA3A		
2.35		CAP2		
2.15		TTF2		
1.99	FN1	FN1	FN1	FN1
1.84	ITGA3			
1.74		PER3		
1.62		NOTCH2	NOTCH2	
1.51		RARB		
1.47		IFNGR1		
1.42		ECE1		
1.41	NET1			
1.35	PIK3CD	PIK3CD	PIK3CD	
1.29	BYSL			
1.25		CTNNB1	CTNNB1	
No. genes >1.5	3	8	2	1
1.5> no. >1.2	3	4	2	0
Total	6	12	4	1

Up SLM8 MS/MA fold	Cell adhesion	Cell commu-nication	Cell adhesion signaling	ECM signaling
7.14		MGP		
5.45	LOXL3			
4.82	TGFBI	TGFBI		TGFBI
4.69		IGFBP5		IGFBP5
3.85		ADAMTS1	ADAMTS1	ADAMTS1
3.64	L1CAM	L1CAM	L1CAM	
3.35		CUBN	CUBN	
3.35	LSAMP			
3.22	LOX			
2.56	CDH1	CDH1	CDH1	
2.53		SEMA3D		
2.52	TNS1			
2.45		SCUBE2	SCUBE2	
2.40	GPC6			
2.34	GLT25D2			
2.28		CORO2B		
2.21		EDIL3		
2.09		LAMB1	LAMB1	LAMB1
2.02		PRELP	PRELP	
1.88		CHRNA6		
1.86		TNC	TNC	
1.85	ITGA5			
1.83		SLC1A1	TSPAN5	
1.80		CD82	CD82	
1.80	PTPRO	PTPRO	PTPRO	
1.77		FABP3		
1.76	NRCAM	NRCAM	NRCAM	
1.70	LOXL2			
1.68		CD70		
1.65	NCAM1	NCAM1	NCAM1	
1.64	HSPG2	HSPG2	HSPG2	HSPG2
1.60	CDH19	CDH19	CDH19	
1.59		LUM	LUM	
1.59		DLL3		
1.59		ECE1		
1.54		TNFRSF19		
1.53		SCG2		
1.50		PTPRJ		
1.50	PTPRM	PTPRM	PTPRM	
1.44	LAMA1	LAMA1		LAMA1
1.42	MAGED2			
1.42	CDON			
1.41		OGT		
1.39	NEO1	NEO1		
1.35		SMAD3		
1.34		GRN		
1.30	ATRNL1			
1.26	TSPAN5	TSPAN5		
1.20	SDC4			
No. genes >1.5	17	31	15	6
1.5> no. >1.2	7	6	1	1
Total	24	37	16	7

Down SLM8 MS/MA fold	Cell adhesion	Cell commu-nication	Cell adhesion signaling	ECM signaling
9.89		TF		
2.40	LOXL4			
1.36		GDF15		
1.28	CHM	CHM		
1.24	CBLL1			
No. genes >1.5	1	1	0	0
1.5> no. >1.2	2	2	0	0
Total	3	3	0	0
