# Supplementary material for: Melanoma Spheroids Grown Under Neural Crest Cell Conditions Are Highly Plastic Migratory/Invasive Tumor Cells Endowed with Immunomodulator Function
Source: PLoS One. 2011 Apr 15;6(4):e18784. doi: 10.1371/journal.pone.0018784 (PMC3078142; doi:10.1371/journal.pone.0018784)
Supplement: Table S5 — Structure/motility regulated genes. List of the genes regulated in melanoma spheroids (MS)/adherent cells (MA) involved in cell structure/motility processes along with their respective fold-changes as determined by biological processes using PANTHER software. (PDF) [file pone.0018784.s010.pdf]

Table S5: Structure/motility regulated genes

Up Mela1 MS/ MA fold	Cell structure and motility	Motility
5.05	JAG1	JAG1
2.75	MET	MET
2.45	GRIP1	
2.31	LCP1	
2.09	KIT	KIT
2.02	CRB1	CRB1
1.83	GSN	
1.47	CORO1C	CORO1C
1.43	MERTK	MERTK
1.39	POR	POR
1.33	KLHL7	
1.25	KLHL20	
No. genes >1.5	7	4
1.5> no. >1.2	5	3
Total	12	7

Down Mela1 MS/MA fold	Cell structure and motility	Motility
2.35	CAP2	
2.34	TES	TES
2.15	TTF2	
1.99	FN1	
1.87	KIF20A	
1.72	WDR63	
1.63	NET1	
1.62	NOTCH2	NOTCH2
1.59	LMNB1	
1.56	DOCK5	DOCK5
1.48	ABLIM3	ABLIM3
1.32	MYO10	
1.32	PLS1	
1.28	FLNB	FLNB
1.20	ARPC1B	
No. genes >1.5	10	3
1.5> no. >1.2	5	2
Total	15	5

Up SLM8 MS/ MA fold	Cell structure and motility	Motility
3.35	CUBN	CUBN
3.15	PDK1	
2.95	FLNB	FLNB
2.92	GEM	
2.52	TNS1	TNS1
2.28	CORO2B	CORO2B
1.87	ITPR1	ITPR1
1.62	KTN1	
1.55	KIAA1598	KIAA1598
1.55	PC	
1.48	KIF21A	
1.39	NEO1	NEO1
1.35	JAG1	JAG1
1.21	LCP1	
No. genes >1.5	10	6
1.5> no. >1.2	4	2
Total	14	8

Down SLM8 MS/MA fold	Cell structure and motility	Motility
2.57	MBP	
2.45	KIF20A	
1.94	GTSE1	
1.66	SVIL	
1.57	TJP1	
1.54	PPP1CA	
1.51	ABL1	ABL1
1.42	DIAPH3	DIAPH3
1.31	ABLIM1	ABLIM1
1.31	CKAP5	
1.29	PHLDB1	
1.28	CHM	
1.28	FLNA	FLNA
1.24	CBLL1	CBLL1
1.23	ACTN4	
1.23	LRRC58	
1.23	ACTR6	
No. genes >1.5	7	1
1.5> no. >1.2	10	4
Total	17	5
