# Supplementary material for: Melanoma Spheroids Grown Under Neural Crest Cell Conditions Are Highly Plastic Migratory/Invasive Tumor Cells Endowed with Immunomodulator Function
Source: PLoS One. 2011 Apr 15;6(4):e18784. doi: 10.1371/journal.pone.0018784 (PMC3078142; doi:10.1371/journal.pone.0018784)
Supplement: Table S6 — Immunity and defense. List of the genes regulated in melanoma spheroids (MS)/adherent cells (MA) involved in immunity and defence processes along with their respective fold-changes as determined by biological processes using PANTHER software. (PDF) [file pone.0018784.s011.pdf]

Table S6: Immunity and defense

Up Mela1 MS/ MA fold	Immunity and defense	Other immune/ defense
41.89	CCL18	
5.05	JAG1	
3.30	TNFRSF19	
2.82	CNIH3	
2.75	MET	
2.57	THBS2	
2.45	GRIP1	GRIP1
2.38	IL6R	
2.23	SDC4	
2.09	KIT	
2.07	SEMA5A	SEMA5A
2.02	CRB1	
1.98	SLC11A2	
1.94	THBS1	
1.71	CD96	
1.71	IFI16	
1.55	IL16	
1.55	DUSP10	
1.48	GAB1	
1.43	MERTK	
1.37	PLSCR1	
1.31	TRAF3	
1.30	TAP2	
No. genes >1.5	18	2
1.5>no.>1.2	5	0
Total	23	2

Down Mela1 MS/MA fold	Immunity and defense	Other immune/ defense
2.07	LRRC40	LRRC40
1.73	PAFAH2	PAFAH2
1.62	NOTCH2	
1.53	IFIT3	
1.49	CREBL1	
1.47	IFNGR1	
1.39	XRCC5	
1.29	PLA2G4A	PLA2G4A
No. genes >1.5	4	2
1.5>no.>1.2	4	1
Total	8	3

Up SLM8 MS/ MA fold	Immunity and defense	Other immune and defense
7.33	CST7	
4.88	PTGS2	
3.82	CNIH3	
3.51	NPTX2	
3.35	CUBN	
2.52	TNS1	
2.12	FCER1G	FCER1G
2.07	IL27RA	
1.87	ITPR1	
1.80	CD82	
1.77	ATM	
1.71	CD55	
1.68	TBXAS1	
1.68	CD70	CD70
1.64	TRAF3	
1.62	ABCC4	
1.55	PC	
1.54	TNFRSF19	TNFRSF19
1.50	NFKBIA	
1.37	RELB	RELB
1.36	NFKB2	NFKB2
1.36	IL16	
1.35	JAG1	
1.34	IRAK3	
1.34	GRN	
1.30	ATRNL1	
1.20	SDC4	
No. genes >1,5	19	3
1,5>no.>1,2	8	2
Total	27	5

Down SLM8 MS/MA fold	Immunity and defense	Other immune/ defense
9.89	TF	
2.61	IFIT3	
2.57	MBP	
2.49	CIITA	
1.94	GTSE1	
1.42	LRRK2	
1.39	ASNA1	
1.24	TRAP1	
1.23	LRRC58	
No. genes >1.5	5	0
1.5>no.>12	4	0
Total	9	0
