# Supplementary material for: Melanoma Spheroids Grown Under Neural Crest Cell Conditions Are Highly Plastic Migratory/Invasive Tumor Cells Endowed with Immunomodulator Function
Source: PLoS One. 2011 Apr 15;6(4):e18784. doi: 10.1371/journal.pone.0018784 (PMC3078142; doi:10.1371/journal.pone.0018784)
Supplement: Table S7 — Molecular functions regulated genes. List of the ECM, cell adhesion, cytoskeleton proteins, actin-binding and non-motor actin-binding regulated genes in melanoma spheroids (MS)/adherent cells (MA) along with their respective fold-changes as determined by molecular functions analysis using PANTHER software. (PDF) [file pone.0018784.s012.pdf]

Table S7: Molecular functions regulated genes

Up Mela1 MS/MA fold	ECM
3.35	LAMA4
2.83	MMP16
2.57	THBS2
2.30	PCOLCE
2.25	HMCN1
2.23	SDC4
2.13	SCUBE2
1.94	THBS1
1.85	LAMC1
1.60	GPR124
1.50	LAMB1
No. genes >1.5	11
1.5> no. >1.2	0
Total	11

Down Mela1 MS/MA fold	ECM
2.91	MME
1.99	FN1
1.34	LRIG2
No. genes >1.5	2
1.5> no. >1.2	1
Total	3

Up SLM8 MS/MA fold	ECM
7.14	MGP
4.52	LGI3
2.45	SCUBE2
2.40	GPC6
2.35	MMP8
2.09	LAMB1
2.02	PRELP
1.86	TNC
1.64	HSPG2
1.60	EDIL3
1.59	LUM
1.55	PC
1.44	LAMA1
1.20	SDC4
No. genes >1.5	12
1.5> no. >1.2	2
Total	14

Down SLM8 MS/MA fold	ECM
2.57	MBP
No. genes >1.5	1
1.5> no. >1.2	0
Total	1

Up Mela1 MS/MA fold	Cell adhesion molecule
15.28	TGFBI
4.58	NCAM1
4.05	TSPAN12
2.97	LSAMP
2.45	GRIP1
2.25	HMCN1
2.23	SDC4
2.14	ITGA7
1.51	ITGA9
No. genes >1.5	9
1.5> no. >1.2	0
Total	9

Down Mela1 MS/MA fold	Cell adhesion molecule
3.83	MCAM
1.99	FN1
1.84	ITGA3
1.35	PIK3CD
1.25	CTNNB1
No. genes >1.5	3
1.5> no. >1.2	2
Total	5

Up SLM8 MS/MA fold	Cell adhesion molecule
4.82	TGFBI
3.64	L1CAM
2.56	CDH1
2.40	GPC6
1.86	TNC
1.85	ITGA5
1.76	NRCAM
1.65	NCAM1
1.60	CDH19
1.42	MAGED2
1.42	CDON
1.39	NEO1
1.26	TSPAN5
1.20	SDC4
No. genes >1.5	9
1.5> no. >1.2	5
Total	14

Down SLM8 MS/MA fold	Cell adhesion molecule
2.40	LSAMP
1.28	CHM
No. genes >1.5	1
Total	1
1.5> no. >1.2	2

Up Mela1 MS/MA fold	Cytoskeletal protein	Actin binding	Non-motor actin binding
2.94	KIF13A		
2.31	LCP1	LCP1	LCP1
2.23	SDC4		
1.99	TTLL6		
1.83	GSN	GSN	GSN
1.47	CORO1C	CORO1C	CORO1C
1.33	KLHL7	KLHL7	KLHL7
1.25	KLHL20	KLHL20	KLHL20
No. genes >1.5	5	2	2
1.5> no. >1.2	3	3	3
Total	8	5	5

Down Mela1 MS/MA fold	Cytoskeletal protein	Actin binding	Non-motor actin binding
2.45	LMO4	LMO4	
2.35	CAP2	CAP2	
2.34	TES	TES	
2.03	KIF2C		
1.87	KIF20A		
1.72	WDR63		
1.66	MYO1B	MYO1B	
1.59	LMNB1		
1.48	ABLIM3	ABLIM3	
1.37	KIFC1		
1.32	MYO10	MYO10	
1.32	PLS1	PLS1	PLS1
1.28	FLNB	FLNB	FLNB
1.25	CTNNB1		
1.20	ARPC1B	ARPC1B	
No. genes >1.5	8	4	0
1.5> no. >1.2	7	5	2
Total	15	9	2

Up SLM8 MS/MA fold	Cytoskeletal protein	Actin binding	Non-motor actin binding
2.95	FLNB	FLNB	FLNB
2.52	TNS1	TNS1	TNS1
2.28	CORO2B	CORO2B	CORO2B
1.86	TNC	TNC	
1.76	TTLL6		
1.62	KTN1		
1.55	KIAA1598	KIAA1598	KIAA1598
1.55	PC		
1.48	KIF21A		
1.30	FHOD1	FHOD1	FHOD1
1.27	CRIP2	CRIP2	
1.25	TTLL5		
1.21	LCP1	LCP1	LCP1
1.20	SDC4		
No. genes >1.5	8	5	4
1.5> no. >1.2	6	3	2
Total	14	8	6

Down SLM8 MS/MA fold	Cytoskeletal protein	Actin binding	Non-motor actin binding
2.45	KIF20A		
2.16	PRC1		
1.88	DLC1		
1.66	SVIL	SVIL	SVIL
1.42	DIAPH3	DIAPH3	DIAPH3
1.31	ABLIM1	ABLIM1	
1.31	CKAP5		
1.29	PHLDB1		
1.28	FLNA	FLNA	FLNA
1.26	MYO1C	MYO1C	
1.23	ACTN4	ACTN4	ACTN4
1.23	LRRC58	LRRC58	
1.23	ACTR6	ACTR6	
No. genes >1.5	4	1	1
1.5> no. >1.2	9	7	3
Total	13	8	4
