# Supplementary material for: Melanoma Spheroids Grown Under Neural Crest Cell Conditions Are Highly Plastic Migratory/Invasive Tumor Cells Endowed with Immunomodulator Function
Source: PLoS One. 2011 Apr 15;6(4):e18784. doi: 10.1371/journal.pone.0018784 (PMC3078142; doi:10.1371/journal.pone.0018784)
Supplement: Table S8 — Molecular functions regulated genes. List of the receptors, other receptors and membrane-bound signaling genes in melanoma spheroids (MS)/adherent cells (MA) along with their respective fold-changes as determined by molecular functions analysis using PANTHER software. (PDF) [file pone.0018784.s013.pdf]

Table S8: Molecular functions regulated genes

Up Mela1 MS/MA fold	Receptor	Other receptor
6.83	DNER	
5.40	PLXNC1	
5.05	JAG1	
4.94	EPHA3	
4.75	DLL3	
4.37	RORB	
4.31	CHRNA6	
3.30	TNFRSF19	
3.21	ERBB4	
2.88	LPHN2	
2.78	SORCS1	SORCS1
2.75	MET	
2.74	UNC13A	UNC13A
2.71	LPHN3	
2.55	P2RX7	
2.38	IL6R	
2.26	SORL1	SORL1
2.25	PTPRZ1	PTPRZ1
2.21	OGFRL1	OGFRL1
2.19	ROBO2	
2.12	P2RY1	
2.09	KIT	
2.02	CRB1	
1.76	ERBB3	
1.71	GRINA	
1.62	EPHA4	
1.60	GPR124	
1.58	PRSS12	PRSS12
1.43	MERTK	
No. genes >1.5	28	6
1.5>nb>1.2	1	0
Total	29	6

Down Mela1 MS/MA fold	Receptor	Other receptor
2.12	SORT1	SORT1
2.00	TFRC	TFRC
1.68	SLC20A1	
1.62	NOTCH2	
1.51	RARB	
1.47	IFNGR1	
1.34	LRIG2	
1.27	LRPPRC	
No. genes >1.5	5	2
1.5>no.>1.2	3	0
Total	8	2

Up SLM8 MS/MA fold	Receptor	Other receptor
5.45	LOXL3	LOXL3
4.52	LGI3	
3.35	CUBN	CUBN
3.22	LOX	LOX
3.14	OLFML3	
2.41	UNC13A	UNC13A
2.22	TRPM3	
2.12	FCER1G	
2.08	RORA	
2.07	IL27RA	
2.05	KDR	
2.02	PRELP	
1.99	EPHA2	
1.88	CHRNA6	
1.87	ITPR1	
1.85	LPHN2	
1.80	PTPRO	PTPRO
1.76	LDLR	LDLR
1.70	VLDLR	VLDLR
1.70	LOXL2	LOXL2
1.64	LRP1	LRP1
1.63	FGFR1	
1.61	SORL1	SORL1
1.59	LUM	
1.59	P2RX7	
1.59	DLL3	
1.54	TNFRSF19	
1.53	LDLRAD3	LDLRAD3
1.52	SEZ6L2	SEZ6L2
1.50	PTPRJ	PTPRJ
1.50	PTPRM	PTPRM
1.48	LRP6	LRP6
1.45	LENG8	
1.40	SORCS1	SORCS1
1.39	NEO1	NEO1
1.37	PLXNC1	
1.35	JAG1	
1.34	IRAK3	
No. genes >1.5	31	14
1.5>no.>1.2	7	3
Total	38	17

Down SLM8 MS/MA fold	Receptor	Other receptor
9.89	TF	
2.78	TRPM1	
2.40	LOXL4	LOXL4
2.34	GPRC5B	
2.34	LRP2	LRP2
1.82	KREMEN1	KREMEN1
1.29	RORB	
nb genes >1,5	6	3
1,5>nb>1,2	1	0
total	7	3

Up Mela1 MS/MA fold	Membrane-bound signaling
6.83	DNER
5.05	JAG1
4.75	DLL3
4.05	TSPAN12
2.82	CNIH3
2.07	SEMA5A
2.02	CRB1
No. genes >1.5	7
1.5> no. >1.2	0
Total	7

Down Mela1 MS/MA fold	Membrane-bound signaling
2.50	SEMA3A
1.62	NOTCH2
No. genes >1.5	2
1.5> no. >1.2	0
Total	2

Up SLM8 MS/MA fold	Membrane-bound signaling
3.82	CNIH3
3.35	CUBN
2.53	SEMA3D
1.80	CD82
1.59	DLL3
1.35	JAG1
1.26	TSPAN5
No. genes >1.5	5
1.5> no. >1.2	2
Total	7

Down SLM8 MS/MA fold	Membrane-bound signaling
No. genes >1.5	0
1.5> no. >1.2	0
Total	0
