# Supplementary material for: Melanoma Spheroids Grown Under Neural Crest Cell Conditions Are Highly Plastic Migratory/Invasive Tumor Cells Endowed with Immunomodulator Function
Source: PLoS One. 2011 Apr 15;6(4):e18784. doi: 10.1371/journal.pone.0018784 (PMC3078142; doi:10.1371/journal.pone.0018784)
Supplement: Table S9 — Signaling pathways regulated genes. List of the genes regulated in melanoma spheroids (MS)/adherent cells (MA) involved in Notch, endothelin and axon guidance signaling along with their respective fold-changes as determined by signaling pathways analysis using PANTHER software. (PDF) [file pone.0018784.s014.pdf]

Table S9: Signaling pathways regulated genes

Up Mela1 MS/ MA fold	Notch
6.83	DNER
5.05	JAG1
4.75	DLL3
1.48	PSEN2
No. genes >1.5	3
1.5> no. >1.2	1
Total	4

Up SLM8 MS/ MA fold	Notch
3.35	CUBN
1.59	DLL3
1.35	JAG1
No. genes >1.5	2
1.5> no. >1.2	1
Total	3

Down Mela1 MS/MA fold	Notch
1.62	NOTCH2
No. genes >1.5	1
1.5> no. >1.2	0
Total	1

Down SLM8 MS/MA fold	Notch
1.67	HEY2
No. genes >1.5	1
1.5> no. >1.2	0
Total	1

Up Mela1 MS/ MA fold	Axon guidance
3.85	DPYSL3
No. genes >1.5	1
1.5> no. >1.2	0
Total	1

Up SLM8 MS/MA fold	Axon guidance
1.79	PLCG1
1.39	NEO1
No. genes >1.5	1
1.5> no. >1.2	1
Total	2

Down Mela1 MS/MA fold	Axon guidance
2.50	SEMA3A
1.41	NET1
No. genes >1.5	1
1.5> no. >1.2	1
Total	2

Down SLM8 MS/MA fold	Axon guidance
1.31	ABLIM1
No. genes >1.5	0
1.5> no. >1.2	1
Total	1

Up Mela1 MS/ MA fold	Endothelin
2.22	ADCY1
2.12	PRKCA
1.57	PRKACB
1.46	MAPK1
No. genes >1.5	3
1.5> no. >1.2	1
Total	4

Up SLM8 MS/ MA fold	Endothelin
4.88	PTGS2
1.93	NPR1
1.87	ITPR1
1.59	ECE1
1.27	NPR2
No. genes >1.5	4
1.5> no. >1.2	1
Total	5

Down Mela1 MS/MA fold	Endothelin
1.51	PIK3R3
1.42	ECE1
1.35	PIK3CD
1.29	PLA2G4A
No. genes >1.5	1
1.5> no. >1.2	3
Total	4

Down SLM8 MS/MA fold	Endothelin
No. genes >1.5	0
1.5> no. >1.2	0
Total	0

Up Mela1 MS/MA fold	Angiogenesis
6.83	DNER
5.05	JAG1
4.94	EPHA3
4.75	DLL3
2.12	PRKCA
1.62	EPHA4
1.46	MAPK1
No. genes >1.5	6
1.5> no. >1.2	1
Total	7

Up SLM8 MS/ MA fold	Angiogenesis
3.35	CUBN
2.05	KDR
1.79	PLCG1
1.63	FGFR1
1.59	DLL3
1.50	PTPRJ
1.35	JAG1
No. genes >1.5	6
1.5> no. >1.2	1
Total	7

Down Mela1 MS/MA fold	Angiogenesis
1.62	NOTCH2
1.51	PIK3R3
1.40	STAT1
1.35	PIK3CD
1.29	PLA2G4A
1.25	CTNNB1
No. genes >1.5	2
1.5> no. >1.2	4
Total	6

Down SLM8 MS/MA fold	Angiogenesis
9.89	TF
2.00	PRKD1
No. genes >1.5	2
1.5> no. >1.2	0
Total	2
