# Supplementary material for: Melanoma Spheroids Grown Under Neural Crest Cell Conditions Are Highly Plastic Migratory/Invasive Tumor Cells Endowed with Immunomodulator Function
Source: PLoS One. 2011 Apr 15;6(4):e18784. doi: 10.1371/journal.pone.0018784 (PMC3078142; doi:10.1371/journal.pone.0018784)
Supplement: Table S10 — Nucleotide sequence of quantitative PCR primers and corresponding annealing temperatures. (PDF) [file pone.0018784.s015.pdf]

Table S10: Nucleotide sequences of quantitative PCR primers and corresponding annealing temperature

Primers	Sequence (5’-3’)	Annealing temperature
OCT4 –F	5’-ACA TCA AAG CTC TGC AGA AAG AAC T-3’	63°C
OCT4 -R	5’-CTG AAT ACC TTC CCA AAT AGA ACC C-3’	
NANOG-F	5’-CAG CTG TGT GTA CTC AAT GAT AGA-3’	63°C
NANOG-R	5’-ACA CCA TTG CTA TTC TTC GGC CAG-3’	
KLF4-F	5’- CGA ACC CAC ACA GGT GAG AA-3’	57°C
KLF4-R	5’- CTG AGC GGG CGA ATT TCC AT-3’	
*SOX*2-F	5’-CAG TCT GCC GAG AAT CCA TG-3’	57°C
*SOX*2-R	5’-TTT TTT TTT TCA GTG TCC ATA TTT C-3’	
