# Supplementary material for: Melanoma Spheroids Grown Under Neural Crest Cell Conditions Are Highly Plastic Migratory/Invasive Tumor Cells Endowed with Immunomodulator Function
Source: PLoS One. 2011 Apr 15;6(4):e18784. doi: 10.1371/journal.pone.0018784 (PMC3078142; doi:10.1371/journal.pone.0018784)
Supplement: Table S11 — Nucleotide sequence of semi-quantitative PCR primers, corresponding annealing temperatures and number of cycles performed. (PDF) [file pone.0018784.s016.pdf]

Table S11: Nucleotide sequences of semi-quantitative PCR primers, corresponding annealing temperature and number of cycles performed

Primers	Sequence (5’-3’)	Annealing temperature	Number of cycles
EPHA2-F	5’-ACC CCC CTC CGC CCC ACA C-3’	62°C	27
EPHA2-R	5’-GCC CTC CAG CCT CAC CTT G-3’		
EPHA3-F	5’-ACT TCC GGG CAG ACA AAG ACC-3’	64°C	27
EPHA3-R	5’-GAC AGG TGA TGG AGC AGC CTG-3’		
ERBB4-F	5-‘GGA GAT AAC CAG CAT TGA GC-*3’*	58°C	35
ERBB4-R	*5’*-TGA TTA AGA GCC ACT AAC ACG-3’		
SEMA3D-F	5’-GGA ATT GTC TCT GAA GCA GCA-3’	58°C	32
SEMA3D-R	5’-TGC GCA AGC TTT CCC ATA AG-3’		
GDF11-F	5’-GAT CCT GGA CCT ACA CGA CTT C-3’	62°C	31
GDF11-R	5’-GGC CTT CAG TAC CTT TGT GAA C-3’		
GDF15-F	5’-CCT CAG AGT TGC ACT CCG AAG-3’	64°C	25
GDF15-R	5’-GAT CCC AGC CGC ACT TCT GG-3’		
PLXC1-F	5’-CTA ACC ATT GCA CTG CAA ACC-3’	60°C	27
PLXC1-R	5’-GAT TCC ATC TTC AAG AAT CAC G-3’		
SNAI2-F	5’-AGA TGC ATA TTC GGA CCC AC-3’	58°C	25
SNAI2-R	5’-CCT CAT GTT TGT GCA GGA GA-3’		
IL-16-F	5’-ATG CCC GAC CTC AAC TCC TC-3’	58°C	28
IL-16-R	5’-CTC CTG ATG ACA ATC GTG AC-3’		
JAG1-F	5’-CTA TGA TGA GGG GGA TGC T-3’	53°C	30
JAG1-R	5’-CGT CCA TTC AGG CAC TGG-3’		
CIITA-F	5’-TTT CTG GGC ACC CGC CTC AC-3’	53°C	30
CIITA-R	5’-CTG GGG GAA GGT GGC TGA GA-3’		
RPL13-F	5’-GGA AGT ACC AGG CAG TGA CAG CT-3’	61°C	22
RPL13-R	5’-CTT CTC GGC CTG TTT CCG TAG-3’		
